# A New *Limnonectes* (Anura: Dicroglossidae) from Southern Thailand

**DOI:** 10.3390/ani11020566

**Published:** 2021-02-22

**Authors:** Siriporn Yodthong, Attapol Rujirawan, Bryan L. Stuart, Anchalee Aowphol

**Affiliations:** 1 Department of Zoology, Faculty of Science, Kasetsart University, Bangkok 10900, Thailand; siri_yodthong@yahoo.com (S.Y.); fsciapr@ku.ac.th (A.R.); 2 Section of Research & Collections, North Carolina Museum of Natural Sciences, Raleigh, NC 27601, USA; bryan.stuart@naturalsciences.org

**Keywords:** Amphibia, *Limnonectes doriae*, bioacoustics, mitochondrial DNA, Southeast Asia

## Abstract

**Simple Summary:**

New species of frogs continue to be discovered at a rapid rate in Southeast Asia, often as a result of reexamining populations of geographically widespread species using new molecular and bioacoustic tools. Here, we show that members of the fanged frog genus *Limnonectes* from Ko Pha-ngan, Ko Samui, and Ko Lanta Yai Islands in southern Thailand can be distinguished from the morphologically similar species *Limnonectes doriae* in molecular, advertisement call, morphometric, and qualitative morphological characters. On the basis of these multiple lines of evidence, we describe the insular populations in southern Thailand as a new species, *Limnonectes pseudodoriae* **sp. nov**. The new species occurs near small streams in low to mid-elevation forests and breeds in terrestrial nests consisting of moist, clay depressions in which the eggs and larvae develop.

**Abstract:**

A new species in the dicroglossid frog genus *Limnonectes* is described from Ko Pha-ngan, Ko Samui, and Ko Lanta Yai Islands in southern Thailand. Males of *Limnonectes pseudodoriae* **sp. nov**. lack a caruncle on top of the head and very closely resemble *L. doriae* (Boulenger, 1887) from Myanmar and western and southern Thailand. However, the new species is distinguished from *L. doriae* and its congeners using an integrative taxonomic approach of morphology, mitochondrial DNA, and bioacoustics. *Limnonectes pseudodoriae* **sp. nov**. differs from *L. doriae* and its congeners by having a unique combination of morphological characters, including body size; skin texture of the interorbital region, dorsum, and shank; toe webbing; relative size of the inner metatarsal tubercle; and coloration of the tympanum, venter, and ova. The advertisement call of the new species is also readily differentiated from that of *L. doriae* in temporal parameters. *Limnonectes pseudodoriae* **sp. nov**. is highly divergent in mitochondrial DNA from *L. doriae* and its congeners, but its phylogenetic position within the genus is not resolved. The natural history of the new species is presented, and the geographic range of *L. doriae* in Thailand is clarified.

## 1. Introduction

The dicroglossid frog genus *Limnonectes* Fitzinger, 1843, contains 76 recognized species and is widely distributed in East and Southeast Asia [1]. Most species in the genus are sexually dimorphic, with males having larger body size, enlarged heads with hypertrophied jaw musculature, and enlarged odontoid processes or ‘fangs” on the lower jaw relative to females. Males of some *Limnonectes* species have a swollen or cap-like structure on the top of the head or “caruncle” [2,3,4,5,6,7,8]. Members of this genus also tend to exhibit a great deal of morphological similarity [9,10]. Many new species of *Limnonectes* have been discovered and described during the last decade, including in Thailand and adjacent regions. *Limnonectes lauhachindai* Aowphol, Rujirawan, Taksintum, Chuaynkern, and Stuart, 2015, from northeastern Thailand morphologically resembled *L. gyldenstolpei* (Andersson, 1916) and *L. dabanus* (Smith, 1922); *L. coffeatus* Phimmachak, Sivongxay, Seateun, Yodthong, Rujirawan, Neang, Aowphol, and Stuart, 2018, from southern Laos morphologically resembled *L. kohchangae* (Smith, 1922); and *L. savan* Phimmachak, Richards, Sivongxay, Seateun, Chuaynkern, Makchai, Som, and Stuart, 2019, from northeastern Thailand and southern and central Laos morphologically resembled *L. dabanus*.

*Limnonectes doriae* (Boulenger, 1887) was originally described from Thagata Juwa, Myanmar. This species is a small to medium-sized *Limnonectes* that lacks a cephalic caruncle, but has broadened convex skin in the interorbital region on top of the head. This species has been reported to occur from Myanmar to western and southern Thailand and Peninsular Malaysia, including the Andaman Islands (India) [1,11,12,13,14,15]. The phylogenetic relationships of *Limnonectes* remain controversial [6,16,17], although several phylogenetic hypotheses (e.g., [16,17,18]) have placed *L. doriae* within a clade containing *L. coffeatus*, *L. hascheanus* (Stoliczka, 1870), *L. kohchangae*, *L. limborgi* (Sclater, 1892), *L. macrognathus* (Boulenger 1917), and *L. plicatellus* (Stoliczka, 1873).

During our fieldwork in 2016–2018, specimens of a small to medium-sized species of *Limnonectes* were collected at three localities in southern Thailand at least 800 air-km southeast of the type locality of *L. doriae*. These specimens generally resembled *L. doriae* in morphology, but differed conspicuously in their advertisement call. To clarify the taxonomic identity of this frog, we reconstruct its phylogenetic relationship in mitochondrial (mt) DNA with other Southeast Asian *Limnonectes* and compare its morphology and advertisement calls to *L. doriae*. Our results provide multiple lines of evidence that support recognizing the southern Thailand *Limnonectes* as a new species.

## 2. Materials and Methods

### 2.1. Sampling

A total of 37 specimens (18 adult males, 15 adult females, and four immatures) of the new species were collected during May 2016 and June–July 2018. Geographical coordinates and elevation were recorded with a Garmin GPSMAP 64s. Ambient air temperature and relative humidity were collected using a Kestrel 4000 Weather Meter. Specimens were collected by hand and humanely euthanized using tricainemethanesulfonate (MS-222; [19]) solution. Liver tissue was removed from each individual, preserved in 95% ethyl alcohol, and stored at −20 °C for molecular analysis. Voucher specimens were initially fixed in 10% buffered formalin and later transferred to 70% ethyl alcohol for long-term preservation. Tissue samples and voucher specimens were deposited in the herpetological collection of the Zoological Museum, Kasetsart University, Bangkok, Thailand (ZMKU).

### 2.2. DNA Extraction, PCR Amplification

Total genomic DNA from 11 individuals of *Limnonectes* sp. and 14 individuals of *L. doriae* (Table S1) were extracted from liver tissue using the NucleoSpin Tissue DNA Extraction Kit (Macherey-Nagel Inc., Düren, Germany). A 913–1180 base pair (bp) fragment of mt DNA that encodes part of the *16S rRNA* gene was amplified by the polymerase chain reaction (PCR; 94 °C 45s, 50–57 °C 30s, 72 °C 1 min) for 35 cycles using at least one of two primer combinations (i) 16S3L [20] and 16Sbr-3′ [21] and/or (ii) L-16S RanaIII [22] and 16Sbr-3′. An 1151–1187 bp fragment of mtDNA that encodes part of the cytochrome c oxidase subunit III gene, the complete tRNA glycine, the complete NADH dehydrogenase subunit 3 gene, and part of the tRNA arginine (*ND3*) was amplified by the polymerase chain reaction (PCR, 94 °C 45s, 49 °C 30s, 72 °C 1 min) for 35 cycles using the primer pair L-COXIII5′ [23] and Arg-HND3 [16]. PCR products were purified using the NucleoSpin Gel and PCR Clean-up Kit (Macherey-Nagel Inc.) and sequenced in both directions on an ABI 3730XL sequencers by Sangon Biotech Inc. (Shanghai, China) using Big Dye version 3 chemistry and the amplifying primers. The internal primers H-16SRana-int [18] and 16Sar-5′ [21] for the first *16S* primer combination, H-16SRanaIII [22] and 16Sar-3′ for the second *16S* primer combination, and H-GlyND3 [23] and L-COXIII [22] for the *ND3* gene were also used in the sequencing reactions. DNA sequences were edited and aligned using Geneious R11 (Biomatter, Ltd., Auckland, New Zealand), and deposited in GenBank under accession numbers MW567806–MW567830 for *16S rRNA* and MW574596–MW574620 for *ND3* (Table S1).

### 2.3. Phylogenetic Analyses

Homologous sequences of all major clades of *Limnonectes* and the outgroups *Fejervarya limnocharis* and *Quasipaa spinosa* (based on [16,17,24,25]) were downloaded from GenBank (Table S1) and aligned to the newly-generated sequences of *Limnonectes* sp. and *L. doriae* using the MUSCLE plug-in as implemented in Geneious R11. We concatenated the *16S rRNA* and *ND3* data together and partitioned the dataset into eight partitions consisting of *16S rRNA*, *COXIII* first, second, and third codon positions, *ND3* first, second, and third codon positions, and *tRNAs*. Phylogenetic relationships were reconstructed using mixed-model Bayesian inference (BI) and maximum likelihood (ML) optimality criterion. The model of sequence evolution that best described each partition was inferred using the Akaike Information Criterion (AIC) as implemented in PartitionFinder2 on XSEDE [26] on the Cyberinfrastructure for Phylogenetic Research (CIPRES; [27]). The selected models were GTR+I+G for *16S rRNA*; SYM+I+G for *COXIII* first codon position; K81UF+I+G for *COXIII* second codon position; TRN+I+G for *COXIII* and *ND3* third codon positions; TVM+I+G for *ND3* first codon position and *tRNAs*; and HKY+I for *ND3* second codon position. The BI analyses were performed using MrBayes v.3.2 on XSEDE [28] on CIPRES. Two independent runs, each with four chains, were run for 20 million generations using the default priors, chain temperature set to 0.1, trees sampled every 1000 generations, and the first 25% of trees discarded as ‘burn-in’. The convergence of the two simultaneous runs, and stationary states of each parameter, were assessed using Tracer v.1.7.1 [29]. Runs were terminated when the effective sample sizes (ESS) of all parameters was ≥200. A 50% majority-rule consensus of the sampled trees was constructed to calculate the posterior probabilities (PP) of the tree nodes. Nodes with PP ≥ 0.95 were considered to be statistically supported. The ML analysis was performed on the IQ-TREE web server [30,31]. The best-fit nucleotide substitution model for each partition followed the BI analysis. Bootstrap analysis was performed using the ultrafast bootstrap approximation [32] with 1000 replicates. Nodes with bootstrap support (BP) values ≥ 95 were considered highly supported [33]. Uncorrected pairwise sequence divergences (*p*-distances) were calculated in MEGA version X [34].

### 2.4. Morphological Measurements

Measurements were taken to the nearest 0.1 mm with digital Vernier calipers (Mitutoyo CD-6ʺ ASX Digimatic Caliper, Japan) and a Nikon SMZ 745 Zoom Stereomicroscope. Fifteen characters were measured on adult specimens following Aowphol et al. [8]:
SVLsnout–vent length;HDLhead length from tip of snout to rear of jaws;HDWmaximum head width;SNTsnout length from tip of snout to anterior corner of eye;EYEeye diameter;IODinterorbital distance;INDinternasal distance;TMPhorizontal diameter of tympanum;SHKshank length;TGHthigh length;LALforearm length, from elbow to base of palmar tubercle;HNDmanus length from tip of third digit to base of palmar tubercle;FTLpes length from tip of fourth toe to base of inner metatarsal tubercle;IMLinner metatarsal tubercle length;IMWinner metatarsal tubercle width.

Sex and maturity were determined by internal examination of gonads and the presence of secondary sexual characteristics (e.g., enlarged odontoid processes, hypertrophied heads, and/or vocal sac openings). The toe webbing formula follows that used by Savage and Heyer [35]. Qualitative characters were measured following Aowphol et al. [8]. Thirty specimens of *L. doriae* were examined from the holdings of ZMKU and FMNH (Data S1; data from FMNH specimens were taken by Somphouthone Phimmachak). Comparative data for males of caruncle-lacking *Limnonectes* species were obtained from their original and expanded descriptions [16,18,36,37,38,39,40].

All morphological measurements (except SVL) were corrected using the allometric equation X_adj_ = X ± β(SVL ± SVL_mean_), where X_adj_ = adjusted value; X = measured value; β = unstandardized regression coefficient of each clade (= species); SVL = measured snout-vent-length; SVLmean = overall average SVL of a given clade (= species) [41,42,43]. Specimens were assigned to group (= species) based on their mtDNA assignment (below). Principal component analysis (PCA) was performed separately by sex using FactoMineR and factoextra package [44,45] in R program v.3.6.1 [46] to assess morphometric differences between groups. Normality of data distribution was tested through the Shapiro-Wilk test and homogeneity of variance was tested by F-test. Morphological differences between species were tested separately by sex using *t*-test (for equal variance) and Welch *t*-test (for unequal variance) if the data conforms normality, and Mann–Whitney U test was used for the non-normal distributed data at a significance level of *p* ≤ 0.05. All statistical analyses were performed in R [46].

### 2.5. Bioacoustic Analyses

Call recordings of *L. doriae* were made at Mae Hong Son Province (Mueang Mae Hong Son District, Mok Champae Sub-district) and of *Limnonectes* sp. at Surat Thani Province (Ko Pha-ngan Island) during June–July 2018. Calls of individual males were recorded at night (1900–2330 h) under natural conditions using a Tascam DR-40 Linear PCM Recorder and a Røde NTG-2 condenser shotgun microphone using uncompressed WAV format at a sampling rate of 44.1 kHz and 16-bit resolution. During recording, the microphone was held 20–30 cm from the calling individual. Each recorded male was subsequently collected and preserved as a voucher specimen. Ambient air temperature was measured to the nearest 1 °C immediately after each recording (within approximately 50 cm of the location of the calling frog) using a Kestrel 4000 Weather Meter.

We analyzed nine calls from one male of *L. doriae* from Mae Hong Son Province and 180 calls (60 calls per individual) of three males of *Limnonectes* sp. from Surat Thani Province. The acoustic analyses were performed in Raven Pro 64 1.5 for Mac (Cornell Lab of Ornithology, Ithaca, NY, USA). The audio spectrograms were achieved using Hanning windows function (frame width with a 512-point fast Fourier transform, 50% overlap, 86.1 Hz resolution) in the spectrum section plot. Spectrogram figures were generated with seewave package version 2.1.4 [47] in R.

Temporal and spectral parameters measured were modified from Cocroft and Ryan [48] and Thomas et al. [49] as follows: Dominant call frequency (the frequency of maximum amplitude measured from a power spectrum generated using Raven’s selection spectrum function over the duration of the entire call); call duration (the time from beginning to end of one call); intercall interval (duration of the interval between consecutive calls); call rate (the total number of calls-1 divided by the time from beginning of first call to beginning of last call); note (the number of notes in a call); note duration (time between the onset and the offset of the middle note in a call); and internote interval (the time interval of the middle notes in a call); note rate (the number of note per second, the total number of notes-1 divided by the time from beginning of first note to beginning of last note). We used call structure description and the terminology of pulses, notes, and calls as defined by Köhler et al. [50] and Emmrich et al. [51]. Comparative advertisement call characters for *L. dabanus*, *L. lauhachindai,* and *L. savan* were taken from the literature [7,8,17].

## 3. Results

### 3.1. Phylogenetic Relationships

**Figure 1:**
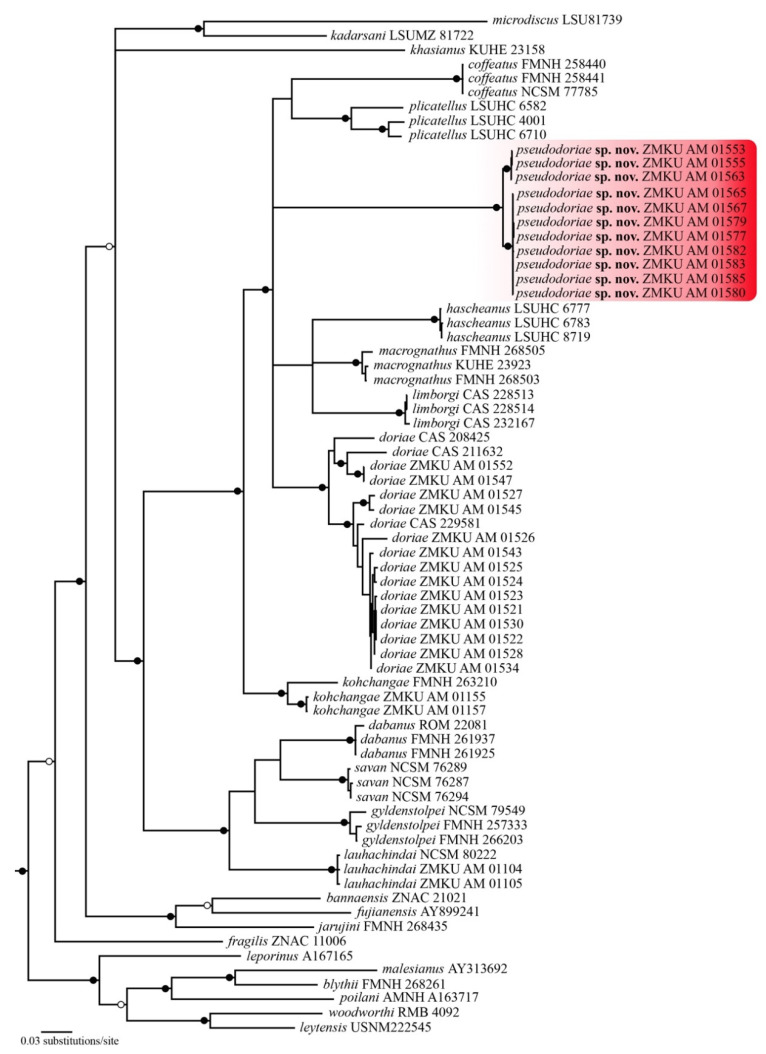
Fifty percent majority-rule consensus phylogram resulting from mixed-model Bayesian analysis of 2380 aligned characters of mitochondrial DNA from dicroglossid frogs in the genus *Limnonectes*. The outgroups *Fejervarya limnocharis* (NC_005055) and *Quasipaa spinosa* (NC_013270) were included in the analysis (not shown). Black circles at nodes indicate Bayesian posterior probabilities ≥ 0.99, and open circle at nodes indicate Bayesian posterior probabilities ≥ 0.95. Numbers at terminal tips are voucher numbers (except those for *L. fujianensis* and *L. malesianus* are GenBank accession numbers). GenBank accession numbers and locality data for sequenced samples are provided in Table S1.

**Figure 2:**
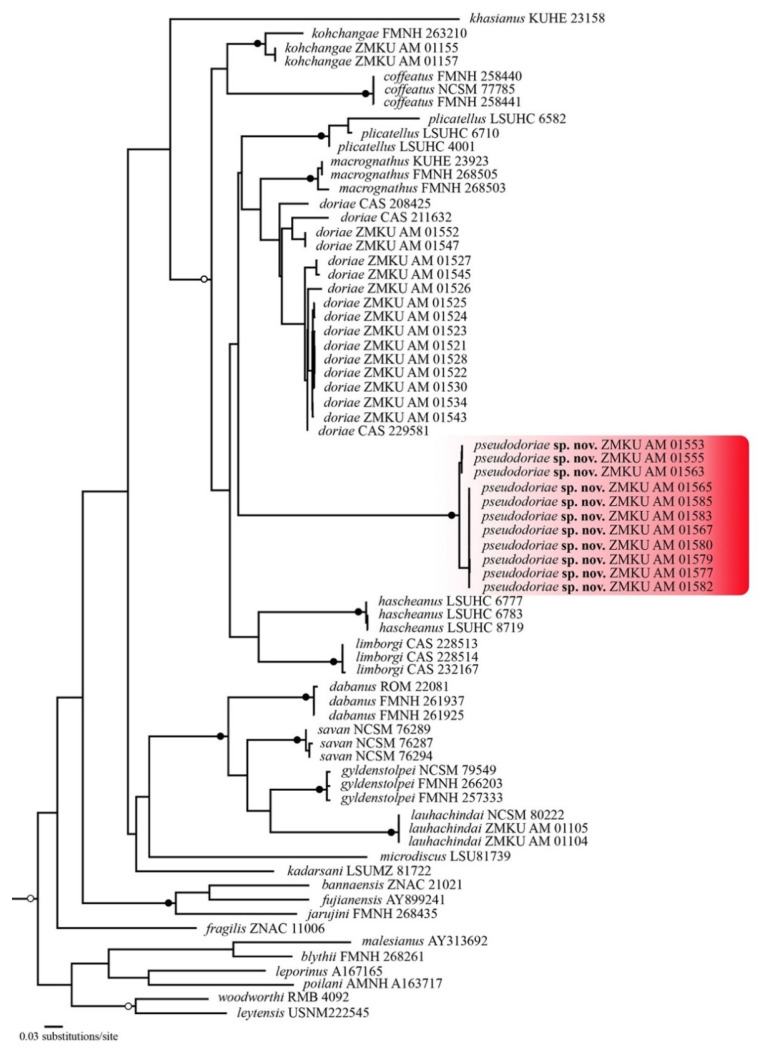
The best tree resulting from maximum likelihood analysis of 2380 aligned characters of mitochondrial DNA from dicroglossid frogs in the genus *Limnonectes*. The outgroups *Fejervarya limnocharis* (NC_005055) and *Quasipaa spinosa* (NC_013270) were included in the analysis (not shown). Black circles at nodes indicate bootstrap values ≥ 0.99, and open circle at nodes indicate boostrap values ≥ 0.95. Numbers at terminal tips are voucher numbers (except those for *L. fujianensis* and *L. malesianus* are GenBank accession numbers). GenBank accession numbers and locality data for sequenced samples are provided in Table S1.

The aligned dataset contained 2380 mtDNA characters from 71 individuals of *Limnonectes* species and two individuals of the outgroup species. The standard deviation of split frequencies was 0.002506 among the two BI runs, and the ESS of all parameters were ≥14,783, indicating that the two runs had been sufficiently sampled and converged. The maximum likelihood value of the best ML tree was lnL = −13,015.988. The 50% majority rule consensus tree from BI analysis and the best ML tree had slightly different topologies, most notably in the phylogenetic position of *L. kohchangae* (Figure 1 and Figure 2, respectively). However, in both analyses, the new species represented a well-supported monophyletic group (1.0 PP, 100 BS) within a clade containing *L. coffeatus*, *L. doriae*, *L. hascheanus*, *L. kohchangae*, *L. limborgi*, *L. macrognathus*, and *L. plicatellus*. Although the exact sister relationship of the new species was unresolved, the new species represented a deeply divergent mtDNA lineage that was distinct from other described species.

The uncorrected *p*-distances within the new species were low, 0.0–1.3%, (mean = 0.6%) for *16S* rRNA, and 0.0–2.0% (mean = 0.9%) for *ND3* (Table 1 and Table 2). The new species had much greater uncorrected *p*-distances from *L. doriae*, 10.6–13.8% (mean = 11.8%), and 17.4–19.3% (mean = 18.7%), and from other congeners, 8.6–17.1% and 19.1–25.3%, in *16S* rRNA and *ND3*, respectively (Table 1 and Table 2).

### 3.2. Morphology

**Figure 3:**
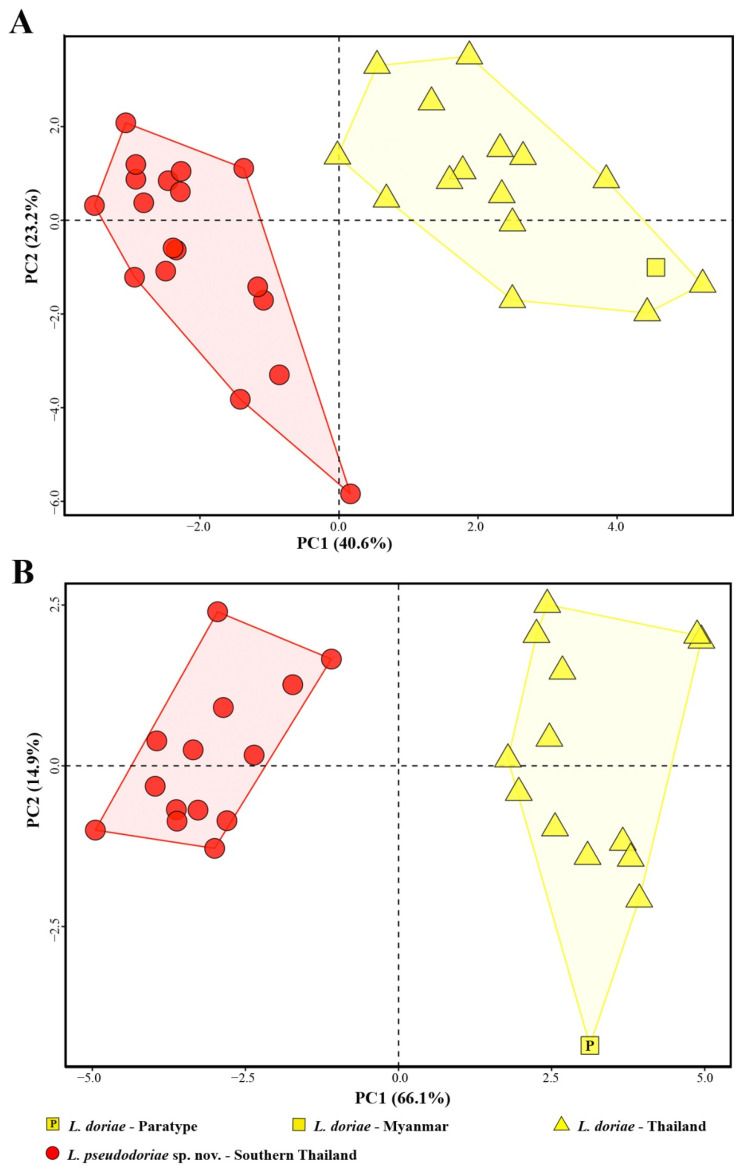
Scatter plot of the first (PC1) and second (PC2) principal components from principal component analysis (PCA) of morphometric measurements of (**A**) males and (**B**) females of *Limnonectes pseudodoriae* **sp. nov**. and *L. doriae*.

PCA of male specimens revealed morphometric differences with no overlap on a plot of the first two axes between the new species and *L. doriae* (Figure 3A). The first two principal components (PC) accounted for a cumulative 63.9% of the total variation and loaded most heavily for EYE_adj_, IND_adj_, SHK_adj_, TGH_adj_, HND_adj_, FTL_adj_, IML_adj_, TMP_adj_, and TMP/EYE along PC1 (40.6% of the total variation), for HDL_adj_, HDW_adj_, SNT_adj_, IOD_adj_, and IMW_adj_ along PC2 (23.2% of the total variation; Table 3). These results indicated that males of the new species had larger tympana (TMP_adj_, TMP/EYE), but smaller eye (EYE_adj_), internarial distance (IND_adj_), forelimbs (HND_adj_), hindlimbs (SHK_adj_, THG_adj_, FTL_adj_), and inner metatarsal tubercles (IML _adj_) than did males of *L. doriae* based on scores of the first two axes (Figure 3A).

PCA of female specimens also indicated morphometric differences with no overlap on a plot of the first two axes between the new species and *L. doriae* (Figure 3B). The first two PC accounted for a cumulative 81.0% of the total variation and loaded most heavily for the most characters except TMP_adj_ and TMP/EYE along PC1 (66.1% of the total variation), for TMP_adj_, and TMP/EYE along PC2 (14.9% of the total variation; Table 3). These results indicated females of the new species had smaller body size (SVL), heads (HDL_adj_, HDW_adj_), snout length (SNT_adj_), internasal distance (IND_adj_), eye (EYE_adj_), interorbital distance (IOD_adj_), forelimbs (HND_adj_, LAL_adj_), hindlimbs (SHK_adj_, TGH_adj_, FTL_adj_), and inner metatarsal tubercles (IML_adj_, IMW_adj_) did than females of *L. doriae* based on scores of the first two axes (Figure 3B).

Statistical comparisons show that most morphological characters were significantly different between the new species and *L. doriae* at *p* < 0.05–0.001 (Table 4). Males of these two species were significantly different in SVL, EYE_adj_, HND_adj_, and TMP_adj_ having *p* < 0.05, and the characters IND_adj_, SHK_adj_, TGH_adj_, FTL_adj_, IML_adj_, IMW_adj_, and TMP/EYE with *p* < 0.001 (Table 4). Females were also significantly different in TMP/EYE having *p* < 0.05, and the most characters, except TMP_adj_, and TMP/EYE with *p* < 0.001 (Table 4). Comparisons of morphometric measurements of adult males and females are given in Tables S2 and S3.

### 3.3. Bioacoustics

**Figure 4:**
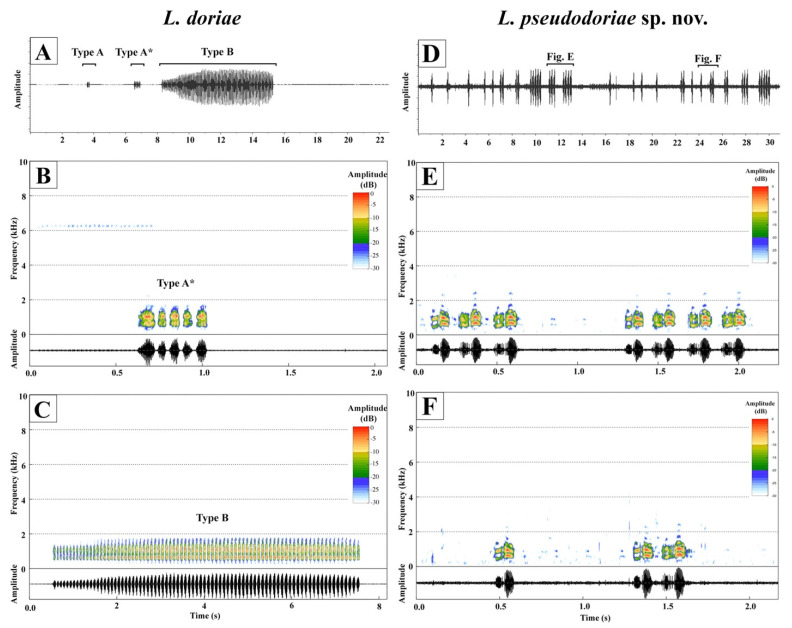
Comparative spectrograms and oscillograms of *Limnonectes doriae* (ZMKU AM 01552; **A**–**C**) and *L*. *pseudodoriae* **sp. nov**. (ZMKU AM 01580; **D**–**F**). (**A**) 22 s waveform of consecutive calls, (**B**) 2 s waveform and spectrogram of Type A call, and (**C**) 8 s waveform and spectrogram of Type B call from *L. doriae*. (**D**) 30 s waveform of consecutive calls, (**E**) 2 s waveform and spectrogram of three-note and four-note calls, respectively, and (**F**) 2 s waveform and spectrogram of one-note and two-note calls, respectively, from *L*. *pseudodoriae* **sp. nov**. Type A * in (**B**) is the enlargement of the Type A call.

*Limnonectes doriae* (ZMKU AM 01552) produced two different call types, when recorded at 24.7 °C, referred to here as Type A (*n* = 6) and Type B (*n* = 3) calls (Figure 4A–C). Type A call is a non-frequency modulated call with uniform notes, consisting of 2–11 non-pulsed notes (5.17 ± 3.43 notes; *n* = 6 calls). The call duration ranged from 151.28 to 820.60 ms (403.08 ± 249.40 ms; *n* = 6 calls). The note duration was 60.88–113.15 ms (75.83 ± 21.13 ms; *n* = 6 notes); the interval between notes was 7.54–14.76 ms (11.05 ± 2.82 ms; *n* = 6 intervals); and note rate was 7.82–13.23 notes per second (11.32 ± 2.02 notes per second; *n* = 6 calls). Dominant frequency ranged from 0.60 to 1.12 kHz (0.92 ± 0.25 kHz; *n* = 6 calls). Type B call is a non-frequency modulated call with uniform notes, and consisted of 81–91 non-pulsed notes (84.67 ± 5.51 notes; *n* = 3 calls). The call duration ranged from 7012.09 to 7714.28 ms (7253.86 ± 398.90 ms; *n* =3 calls). The note duration was 72.56.88–77.62 ms (75.58 ± 2.67 ms; *n* = 3 notes); the interval between notes was 8.48–15.37 ms (11.44 ± 3.55 ms; *n* = 3 intervals); and note rate was 11.54–11.77 notes per second (11.65 ± 0.11 notes per second; *n* = 3 calls). Dominant frequency ranged from 0.69 to 1.12 kHz (0.98 ± 0.25 kHz; *n* = 3 calls). The advertisement calls of *L. doriae* were repeated at a rate of approximately 0.56 calls per minute (*n* = 1 individual), and the silent interval between calls varied from 0.41–451.06 s (108.22 ± 189.88 s; *n* = 8 intervals). Each note of these calls showed weak or no harmonics and no frequency modulation.

The advertisement call of *Limnonectes* sp. from Ko Pha-ngan Island (Figure 4D–F) is based on calls of three males (ZMKU AM 01579–81) recorded at 26.0–26.1 °C. The advertisement call is a non-frequency modulated call with uniform notes, consisting of 1–10 notes (1.98 ± 1.62 notes; *n* = 180 calls) with a call duration ranging from 124.56 to 1965.07 ms (335.66 ± 333.62 ms; *n* = 180 calls). Notes are composed of two undistinguished pulses with a note duration of 117.65–183.86 ms (139.94 ± 12.69 ms; *n* = 180 calls); interval between notes 7.97–75.05 ms (41.42 ± 11.94 ms; *n* = 96 intervals); and note rate of 4.55–7.11 notes per second (5.60 ± 0.47; *n* = 96 calls). Dominant frequency ranges from 0.52 to 1.03 kHz (0.89 ± 0.13 kHz; *n* = 180 calls). The advertisement calls of *Limnonectes* sp. were repeated at a rate of approximately 32.46–46.39 calls per minute (37.65 ± 7.62 call per minute); and the silent interval between calls varies from 0.38–10.84 s (1.28 ± 1.52 s; *n* = 179 intervals). Each note of the calls showed two distinct harmonic bands with weak or without frequency modulation. Acoustic parameters of the new species are summarized in Table 5 and Table S4.

### 3.4. Systematic Account

The samples of *Limnonectes* from the three localities in southern Thailand differed from *L. doriae* and congeners in mtDNA, morphology, and advertisement call. On the basis of these three corroborated lines of evidence, the southern Thailand *Limnonectes* is hypothesized to be a distinct species, and is described as new as follows.

**Figure 5:**
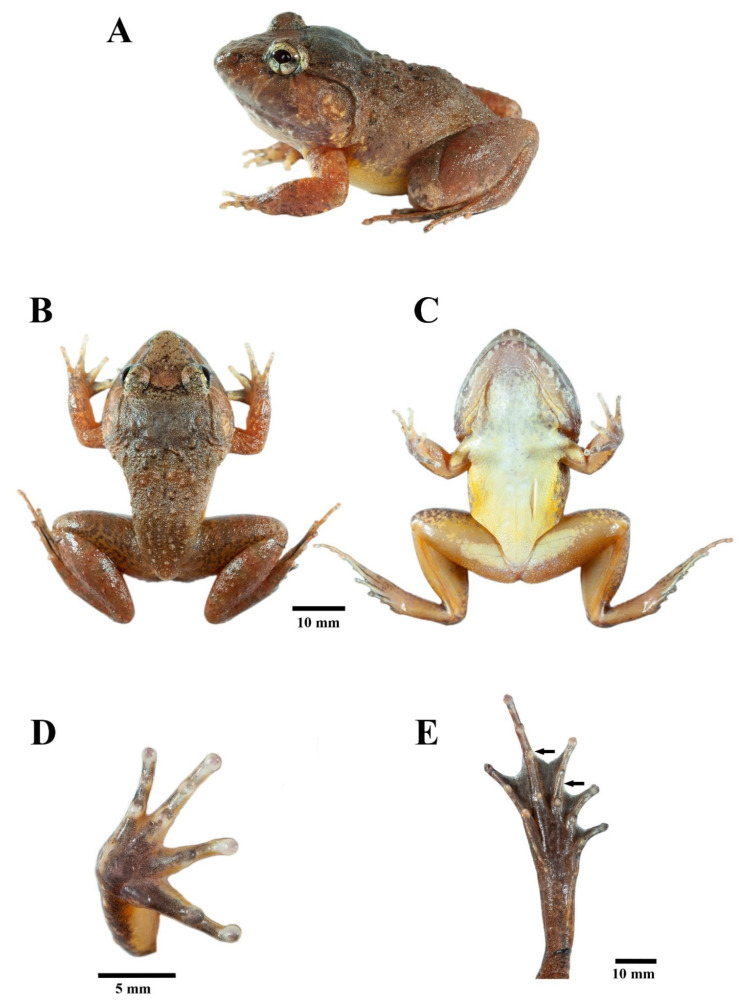
Holotype of *Limnonectes pseudodoriae* **sp. nov**. (ZMKU AM 01567) in life. (**A**) Lateral view, (**B**) dorsal view, (**C**) ventral view, (**D**) palmar view of the right hand, and (**E**) plantar view of the right foot. Arrows refer to diagnostic characters in toe webbing.

*Limnonectes pseudodoriae* **sp. nov**. (Figure 5).

http://zoobank.org/urn:lsid: zoobank.org:pub:E7C22C61-774A-49C3-9355-32F88C340B96.

Holotype: ZMKU AM 01567 (field no. AA 06430), adult male (Figure 5), collected from Thailand, Surat Thani Province, Ko Pha-ngan District, Ko Pha-ngan Sub-district, Pheang Waterfall (9°44.086’ N, 100°00.826’ E, 44 m elevation), 17 June 2018, by Siriporn Yodthong, Attapol Rujirawa, Natee Ampai, and Korkhwan Termprayoon.

Paratypes: Three adult males (ZMKU AM 01565–66, ZMKU AM 01568) and five adult females (ZMKU AM 01569–73), same data as holotype. Two adult males (ZMKU AM 01574–75) and one adult female (ZMKU AM 01576), same data as holotype except Khao Tha Nob Weir (9°44.994’ N, 100°0.801’ E, 105 m elevation). Four adult males (ZMKU AM 01578–81) and one adult female (ZMKU AM 01577), same data as holotype except Mueang Mak Mine (9°44.301’ N, 100° 01.989’ E, 322 m elevation), 18 June 2018. Three adult males (ZMKU AM 01582–84) and five adult females (ZMKU AM 01585–89), collected from Thailand, Surat Thani Province, Ko Samui District, Ang Thong Sub-district, Song Reau Waterfall (9°32.755’ N, 99°57.612’ E, 85 m elevation) on 20 July 2018 by Siriporn Yodthong, Attapol Rujirawa, Natee Ampai, and Korkhwan Termprayoon. Five adult males (ZMKU AM 01553, ZMKU AM 01555, ZMKU AM 01558, ZMKU AM 01561, ZMKU AM 01563) and three adult females (ZMKU AM 01559–60, ZMKU AM 01562), collected from Thailand, Krabi Province, Ko Lanta District, Ko Lanta Yai Sub-district, Tham Khao Mai Kaew Cave (7°32.057’ N, 99°04.220’ E, 105 m elevation) on 3 May 2016 and 11 June 2017 by Siriporn Yodthong, Attapol Rujirawan, Natee Ampai, and Piyawan Puanprapai.

Referred specimens: One immature specimen (ZMKU AM 01564), same data as holotype except Paradise Waterfall (9°46.683’ N; 100°00.539’ E, 48 m elevation), 16 June 2018. Three immature specimens (ZMKU AM 01554, ZMKU AM 01556–57) collected from Thailand, Krabi Province, Ko Lanta District, Ko Lanta Yai Sub-district, Tham Khao Mai Kaew Cave (7°32.057’ N, 99°04.220’ E, 105 m elevation) on 3 May 2016 by Siriporn Yodthong, Attapol Rujirawa, and Natee Ampai.

Etymology: The specific name *pseudodoriae* is a noun in apposition. The Greek word pseudo means “false” or “resembling”, in reference to the morphological similarity between the new species and *L. doriae*. 

Suggested common names: False Doria’s Fanged Frog (English), Kob Tam Than Lueang กบตามธารเหลือง (Thai).

#### 3.4.1. Diagnosis

*Limnonectes pseudodoriae* **sp. nov**. is assigned to the genus *Limnonectes* on the basis of its inferred phylogenetic position (Figure 1 and Figure 2), the presence of fang-like odontoid processes on the lower jaw [6,10], and enlarged heads with hypertrophied jaw musculature in adult males [6]. This species can be distinguished from its congeners by having the following combination of characters: (1) Small to medium body size, with SVL of adult males 42.6–48.2 mm (45.2 ± 1.7, *n* = 18), of adult females 36.0–44.1 mm (40.0 ± 2.8, *n* = 14); (2) males lacking cephalic caruncles, but having broadened convex skin with densely grained translucent spinules on the interorbital region from the level of the posterior margin of the eyes to the level of the posterior margin of the tympanum; (3) males with enlarged heads with hypertrophied jaw musculature; (4) two enlarged odontoid processes, triangular, sharp-tipped, angled posteriorly on anterior margin of the lower jaw, larger in adult males than females; (5) tympanum dark brown with yellow mottling, horizontal diameter in adult males greater than that of the eye, in juveniles and females less than that of the eye; (6) dorsum with enlarged, oval tubercles, and slightly elongated tubercles, not arranged in rows; (7) dorsal surface of shank covered with small, distinct, moderately dense, homogenously-sized tubercles tipped with translucent spinules; (8) chin and throat yellowish white with dark brown or gray mottling (9) chest, belly, ventral surface of upper forelimb and groin yellowish white; (10) ventral surfaces of forelimbs and hindlimbs orange-yellow; (11) Toe V with raised, but unmovable dermal ridge along outer margin; (12) inner metatarsal tubercle length approximately 35% that of Toe I; (13) toe webbing formula: I0−2II0−2½III0−(2½-3)IV(2½-3)−0V; and (14) ova with pigmented poles.

#### 3.4.2. Description of Holotype

Habitus moderately stocky; body tapering to groin. Head broad and depressed; head width slightly greater than head length. Snout obtusely pointed in dorsal view, rounded in profile, projecting well beyond lower jaw in profile; nostril dorsolateral, below canthus, much closer to tip of snout than eye; internarial distance less than interorbital distance; canthus rostralis indistinct, rounded, slightly constricted behind nostrils; lores concave, oblique; eye diameter 51% of snout length, interorbital distance greater than upper eyelid width; pineal ocellus visible; tympanum round, not elevated from side of head, annulus visible, tympanum diameter approximately 149% eye diameter and greater than distance between tympanum and eye; small, slit-like vocal sac openings on anterior floor of mouth near lateral margin of tongue; vomerine teeth on two short oblique ridges, equal in distance to each other as choanae, beginning on anterior inner edge of choanae and extending toward back; two enlarged odontoid processes, triangular, angled posteriorly on anterior margin of lower jaws; median triangular protuberance at mandibular symphysis.

Forelimbs moderately robust. Fingers moderately slender, without webbing; tips of finger rounded, weakly expanded into discs; relative finger lengths II = IV < I < III; subarticular tubercles rounded, distinct, one on Finger I−II, two on Finger III−IV; thenar tubercle oval, distinct; two distinct oval palmar tubercles in contact at base of Finger III−IV; nuptial pad absent.

Hindlimb moderately robust. Toe moderately slender; tips of toes rounded, expanded into small discs; relative toe lengths I < II < V < III < IV; toe webbing formula I0−2II0−2½III0−3IV3−0V, webbing on Toe I to base of disc, on preaxial side of Toe II to level of subarticular tubercle and continuing as a fringe to base of tip, on proximal side of Toe II to base of tip, on preaxial side of Toe III to midway between distal and proximal subarticular tubercle and continuing as a fringe to base of tip, on postaxial side of Toe III to base of tip, on preaxial and postaxial sides of Toe IV to level of middle subarticular tubercle and continuing as a fringe to base of tip, and on Toe V to base of tip; raised but immovable dermal ridge along the outer side of the Toe V from base of tip to level of outer metatarsal tubercle; subarticular tubercles prominent; distinct fold on distal two-thirds of tarsus; distinct, elongate, oval, inner metatarsal tubercle, length approximately 35% distance between tip of Toe I and tubercle; no outer metatarsal tubercle.

Skin on snout and side of head shagreened; upper eyelid covered with rounded tubercles; dorsum shagreened covered with enlarged, oval tubercles, and slightly elongated tubercles, most tubercles condensed near flank and lower back; tubercles tipped with single, translucent spinules; caruncle absent, but broadened convex skin with densely grained translucent spinules on interorbital region from level of the posterior margin of the eyes to level of posterior margin of the tympanum; tubercular ridges from posterior margin of eyelid to occiput forming indistinct W-shape; dorsolateral fold absent; distinct supratympanic ridge from posterior corner of eye to axilla; skin on dorsal surfaces of forelimbs and thigh shagreened; small, distinct, moderately dense, homogenously-sized tubercles tipped with translucent spinules on dorsal surfaces of shank; skin on throat wrinkled, that on remaining ventral surfaces smooth; rictal gland absent.

#### 3.4.3. Color of Holotype in Life

Dorsum dark orange brown; W-shaped tubercular ridge and tubercles on upper back with scattered dark brown mottling; snout beige with scattered dark brown mottling; dark brown band between eyes with beige posterior margin; broadened convex skin on interorbital region dark brown; dark brown band under canthus and supratympanic fold, extending from nostril to upper half of tympanum; tympanum dark brown with yellow mottling; upper surfaces of flank dark orange brown with darker mottling; lower surfaces of flank dark yellow. Upper lip orange brown with white mottling alternating with broad dark brown bands, lower lip dark brown with white mottling. Iris bronze with black, vermiform mottling and horizontal bars forming +-shaped marking over eye. Dorsal surfaces of limb with indistinct dark brown crossbands. Chin creamy white and throat yellowish, with gray-brown mottling; chest creamy white; ventral surfaces of upper arm, belly, and upper groin with yellowish white, ventral surfaces of lower arm and hindlimb with yellow orange; ventral surfaces of hands and feet uniformly dark brown (Figure 5).

#### 3.4.4. Coloration of Holotype in Preservative

**Figure 6:**
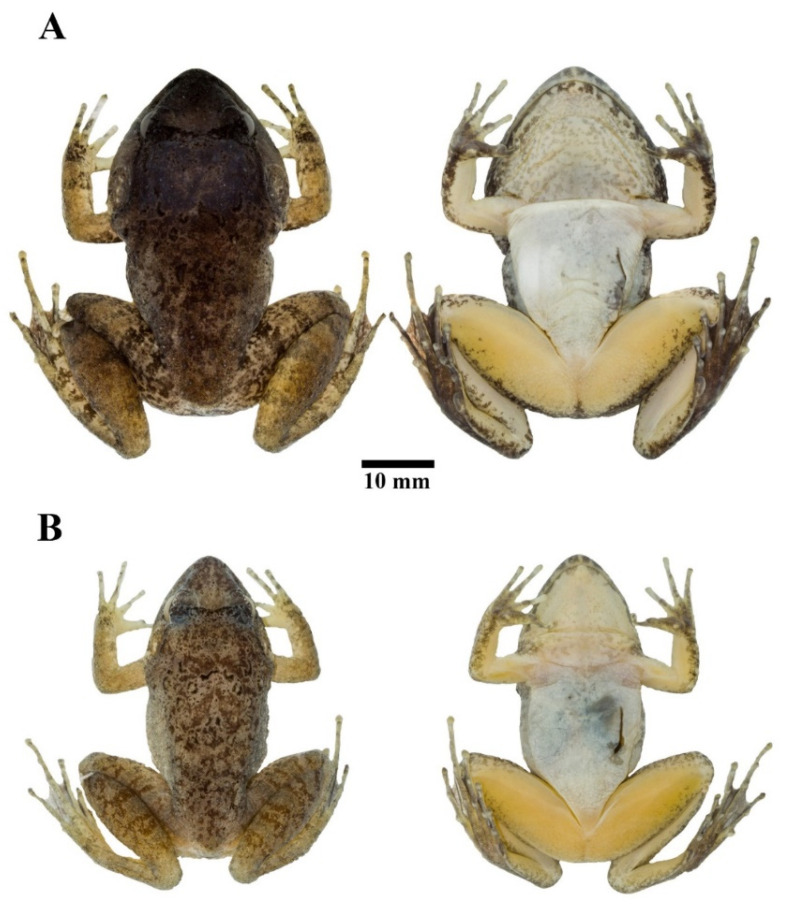
Sexual dimorphism of *Limnonectes pseudodoriae* **sp. nov**. in preservative. Dorsal (left) and ventral (right) views of (**A**) holotype male (ZMKU AM 01567), and (**B**) paratype female (ZMKU AM 01573).

Dorsum nearly uniformly brown with indistinct, scattered dark brown mottling, lips with indistinct dark brown broad bands, and dorsal surfaces of limbs with indistinct dark brown cross-bands. Tympanum and forelimbs with lighter brown than remaining dorsum. Interorbital band distinct dark brown. Tubercles on dorsum encircled with dark brown. Chin and throat with dark brown mottling, belly cream, groin yellowish white, and ventral part of hands and feet uniformly dark brown (Figure 6A).

#### 3.4.5. Morphological Variation

Females lack broadened convex skin on interorbital region; have smaller body size than males; have narrower heads in dorsal view than males (Figure 6); have relatively smaller tympana than males, with the horizontal diameter of tympanum less than that of eye; have smaller and shorter odontoid processes than males; have less distinct and fewer tubercles on dorsum, flank, and posterior region of shank than males; lack wrinkled skin on the throat; have less mottling on the throat and chin than males; and have ova with pigmented poles.

**Figure 7:**
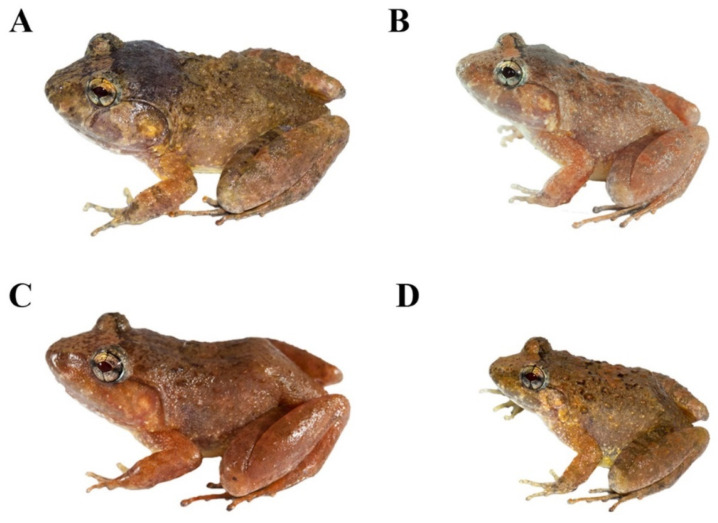
Paratypes of *Limnonectes pseudodoriae* **sp. nov**. (**A**) adult male (ZMKU AM 01568), (**B**) adult male (ZMKU AM 01575) from Ko Pha-ngan Island. (**C**) Adult male (ZMKU AM 01555) from Ko Lanta Yai Island and (**D**) adult female (ZMKU AM 01573) from Ko Pha-ngan Island.

The largest male in the type series is paratype ZMKU AM 01578 having SVL of 48.2 mm, the second largest is paratype ZMKU AM 01579 having SVL of 48.1 mm, and the third largest is the holotype (Table S2). The degree of toe webbing on the preaxial and postaxial sides of Toe IV varies in some specimens by reaching to midway between the distal and proximal subarticular tubercles before continuing as a fringe to base of tip. Some specimens have more or less mottling on the dorsum, less mottling on the throat, more distinct crossbands on the dorsal surfaces of limbs, and less distinct lip bands than the holotype (Figure 7). Morphological measurements of the type series are summarized in Tables S2 and S3.

#### 3.4.6. Habitat, Distribution, and Natural History

**Figure 8:**
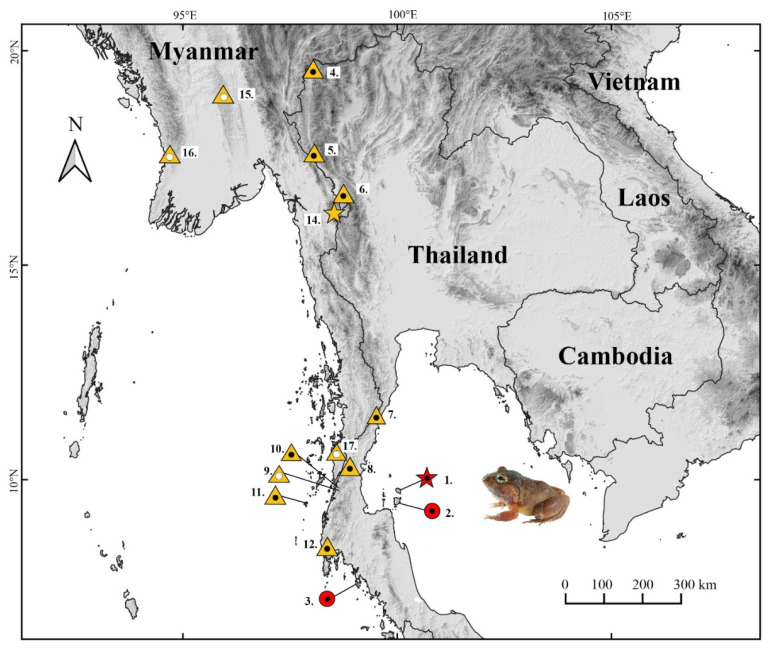
Map showing the type locality (red star) at Ko Pha-ngan Island, Surat Thani Province, Thailand and paratype localities (red circles) at Ko Samui Island, Surat Thani Province and Ko Lanta Yai Island, Krabi Province, Thailand of *Limnonectes pseudodoriae* **sp. nov**., the type locality (yellow star) in Myanmar, and the localities of studied specimens (yellow triangles) of *L. doriae* in Thailand and Myanmar. Numbers correspond to those in Table S1. Shaded symbols indicate morphological data only, Shaded symbols with white center dot indicate molecular data only, and shaded symbols with black center dots indicate both molecular and morphological data were studied.

*Limnonectes pseudodoriae* **sp. nov**. is currently known from only three localities in southern Thailand: Ko Pha-ngan Island (type locality) and Ko Samui Island in the Gulf of Thailand, and Ko Lanta Yai Island in the Andaman Sea (Figure 8). The new species occurs in hill and semi-evergreen forests from 44–322 m elevation, including secondary forests surrounded by agricultural lands (orchards and rubber plantation) and human dwellings. Specimens (*n* = 37) were collected at night (1900–2230 h) during the early–mid rainy season (May–July) on the forest floor (35.1%; *n* = 13); in small (≤8 cm wide) clay holes (8.1%; *n* = 3) associated with a permanent, small (1–4 m wide) stream; on rocky and sandy banks (37.8%; *n* = 14) of a small, rocky stream; in puddles (16.2%; *n* = 6); or on small (≤15 cm tall) shrubs (2.7%; *n* = 1).

**Figure 9:**
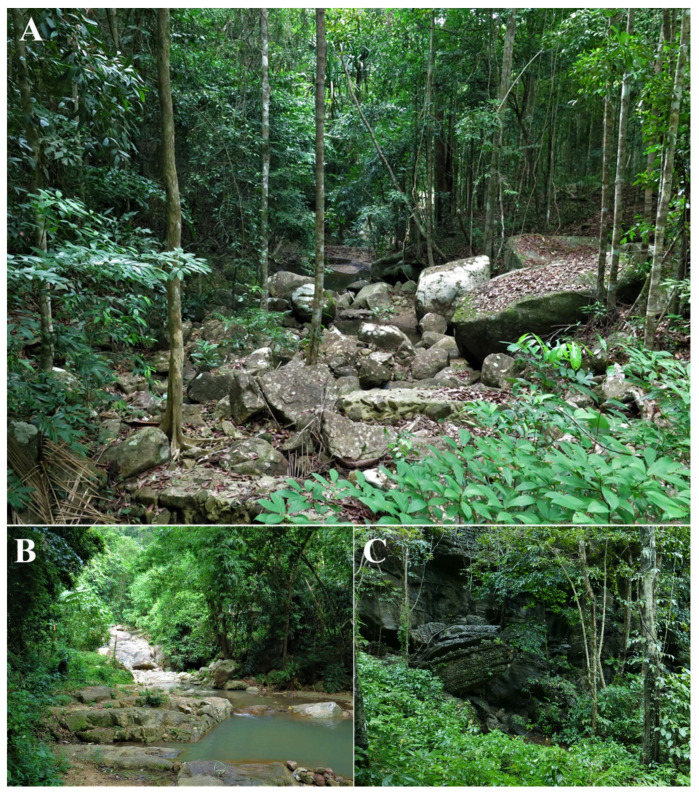
Sampling localities of *Limnonectes pseudodoriae* **sp. nov**. at (**A**) the type locality in Pheang Waterfall, Ko Pha-ngan Island, Surat Thani Province, (**B**) Song Reau Waterfall, Ko Samui Island, Surat Thani Province, and (**C**) Tham Khao Mai Kaew Cave, Ko Lanta Yai, Krabi Province.

**Figure 10:**
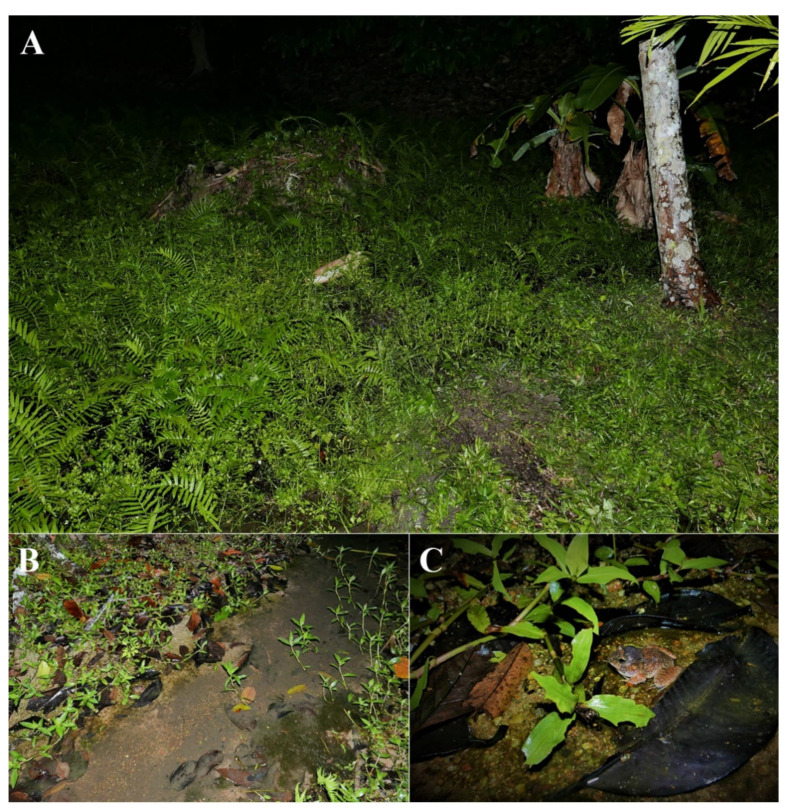
Habitat of calling males of *Limnonectes pseudodoriae* **sp. nov**. (**A**,**B**) An orchard near the forest edge in Ko Pha-ngan Island, Surat Thani Province, and (**C**) an adult male in shallow water, presumably where they were heard calling.

At Ko Pha-ngan Island, the holotype was found while calling on the stream bank of Pheang Waterfall (Figure 9A) during a drizzle rain on 17 July 2018. Other calling males (ZMKU AM 01565, ZMKU AM 01568) and females (not collected) were found in a shallow puddle that was covered with grass in the same area, but these calls were not recorded. Advertisement calls were recorded from two males in a small clay hole under a clump of ferns (ZMKU AM 01579–80) and one male (ZMKU AM 01581) in a small, shallow (<5 cm deep), flowing stream with gravel and clay that was covered with grasses, weeds, and leaf litter during a drizzle rain (Figure 10) in an orchard near the forest edge of Mueang Mak Mine on 18 July 2018. More than 15 adult males were calling in the same area (ca. 4 m around), but not collected. All collected female specimens were gravid, with many bicolored eggs visible inside the abdominal cavity through the belly skin. Other sympatric frog species found at this locality included *Duttaphrynus melanostictus* (Schneider, 1799), *Fejervarya limnocharis* (Gravenhorst, 1829), and *L. blythii* (Boulenger, 1920).

**Figure 11:**
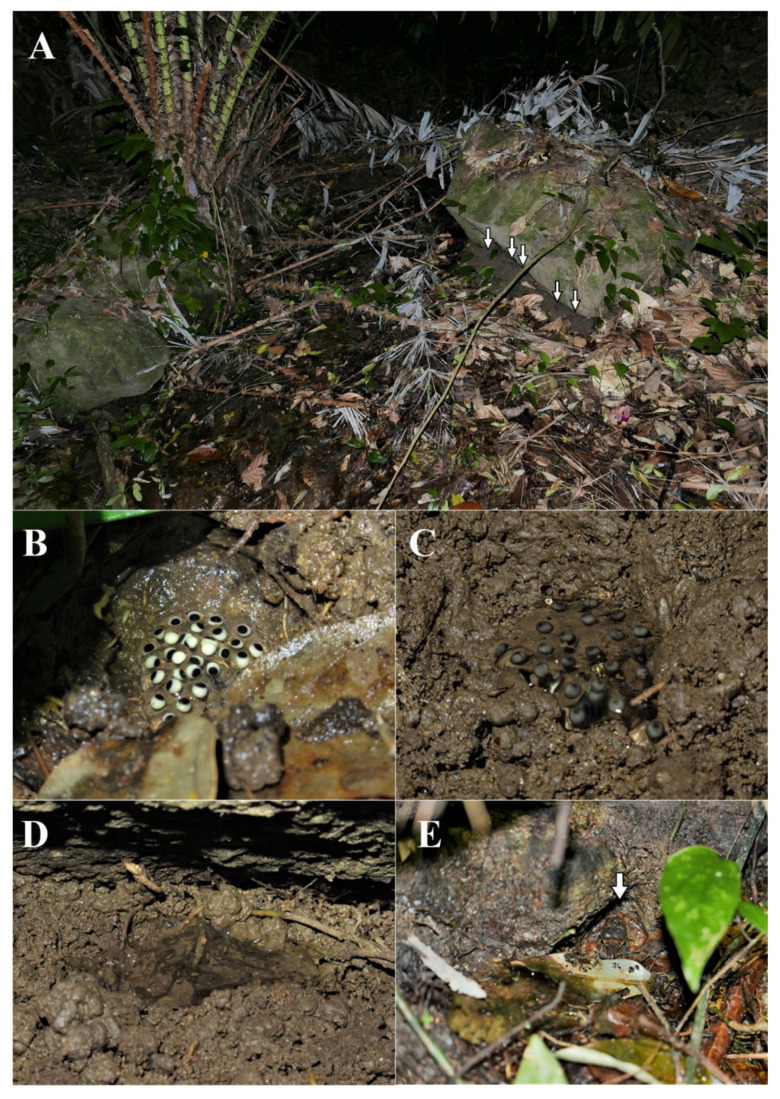
(**A**) Oviposition site of *Limnonectes pseudodoriae* **sp. nov**. at Song Reau Waterfall, Ko Samui Island, Surat Thani Province, (**B**) clutch of 30, bicolored eggs in a nest without free water, (**C**) clutch of 25 developing eggs, (**D**) 16 tadpoles in a small volume of fluid, and (**E**) adult male near the oviposition site (not collected).

At Ko Samui Island, the oviposition site of *L. pseudodoriae* **sp. nov**. was found at Song Reau Waterfall on 20 July 2018 (Figure 9B). The eggs were deposited in terrestrial nests on the forest floor, under boulders next to a small, shallow, flowing stream (ca. 1 m wide) at the edge of secondary forest (Figure 11). This site was approximately 5 m away from the main waterfall stream. The egg clutches were laid in a moist, shallow, small clay hole that was covered with leaf litter. We observed eight isolated nests in the same area (1 m wide) at an ambient temperature of 28.1 °C and relative humidity 84.4%. The nests were approximately 5–7 cm in diameter and 3–5 cm deep. The nests contained 17, 18, 19, 25, and 30 eggs (not collected), and 12, 16, and 21 tadpoles (not collected) in the moist clay without free water (Figure 11B,C) or only a small volume of fluid (Figure 11D). The recently oviposited eggs were bicolor, with the animal hemisphere dark and the vegetal hemisphere white in color. Each egg was enclosed in a large volume of a clear jelly layer that melded with that of other eggs. When the egg develops, the dark pigmentation is more evenly distributed at its surface (Figure 11B,C). Tadpoles remained relatively motionless and did not exhibit apparent feeding behavior during their observation at night (Figure 11D). Moreover, we also found one calling male (ZMKU AM 01583), two non-calling adult males (ZMKU AM 01582, ZMKU AM 01584), and other unvouchered males surrounding the nests (≤1 m from the nest; Figure 11E). All collected female specimens were gravid, with many bicolored eggs visible inside the abdominal cavity through the belly skin. Other sympatric frog species found at this locality included *L. blythii* and *Sylvirana nigrovittata* (Blyth, 1856).

At Ko Lanta Yai Island, *L. pseudodoriae* **sp. nov**. was taken on the forest floor covered with grasses and leaf litter, or on a gravel and sand bank, near a small, shallow, flowing stream (ca. 1 m wide) near Thum Khao Mai Kaew Cave on 3 May 2016 and 11 July 2017 (Figure 9C). No calling males were observed. Other sympatric frog species found at this locality included *D. melanostictus* (Schneider, 1799), *F. limnocharis* (Gravenhorst, 1829), *Kaloula pulchra* Gray, 1831, *L. blythii*, *Microhyla butleri* Boulenger, 1900, *M. mukhlesuri* Hasan, Islam, Kuramoto, Kurabayashi, and Sumida, 2014, *M. heymonsi* Vogt, 1911, and *Polypedates leucomystax* (Gravenhorst, 1829).

#### 3.4.7. Comparisons

*Limnonectes pseudodoriae* **sp. nov**. differs from all other species of *Limnonectes* that occur in mainland Southeast Asia, except *L. coffeatus*, *L. doriae*, and *L. kohchangae*, by having the combination of adult males lacking a cephalic caruncle (sensu [6]), small to medium body size with SVL 30–50 mm in adults (Table S2), and a visible tympanum.

*Limnonectes pseudodoriae* **sp. nov**. differs from *L. coffeatus* and *L. kohchangae* by having SVL 42.6–48.2 mm in adult males (SVL 37.9 mm in *L. coffeatus* [18]); broadened convex skin on the interorbital region, extending from the level of the posterior margin of the eye to the level of the posterior margin of the tympanum (absent in *L. coffeatus* and *L. kohchangae*); tympanum dark brown with yellow mottling (distinctly bi-colored with the upper half heavily pigmented in *L. kohchangae*; uniformly colored without distinct dark markings on upper half in *L. coffeatus*); an unmovable dermal ridge along the outer side of the Toe V (movable in *L. kohchangae* and *L. coffeatus*); and by having toe webbing formula I0−2II0−2½III0−(2½-3)IV(2½-3)−0V (vs. I0−2II0−2III0−3IV3−0V in *L. coffeatus*; I0−1½II0−2III0−3IV3−0V in *L. kohchangae*).

**Figure 12:**
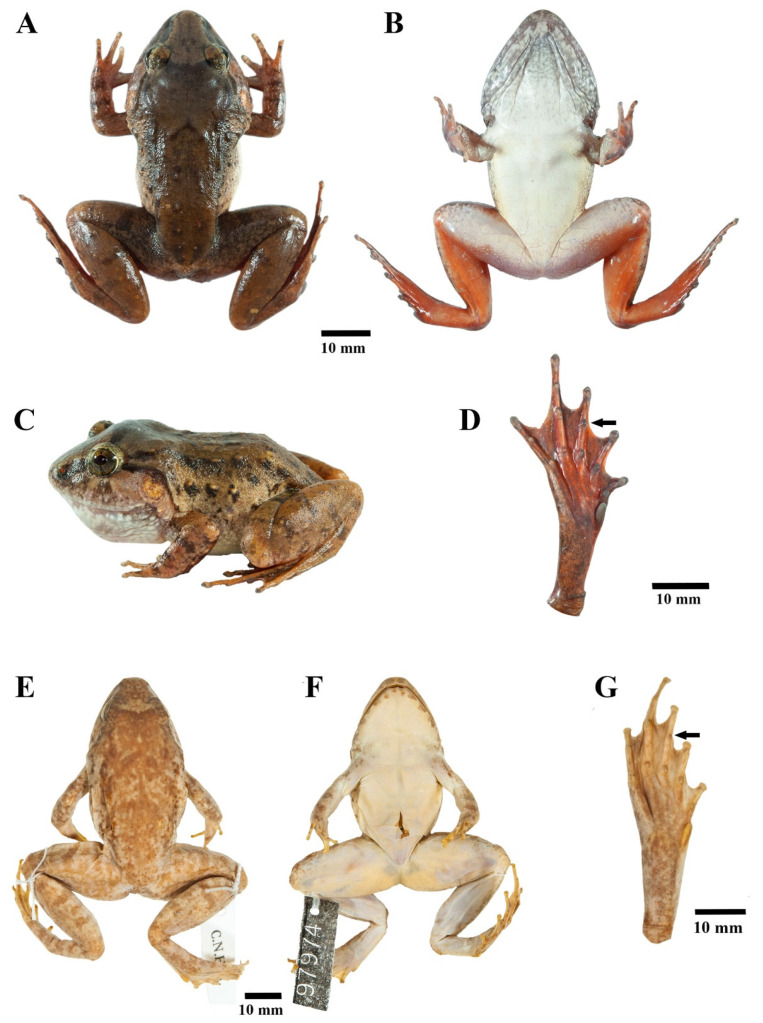
Adult male of *Limnonectes doriae* in life at Mok Champae Sub-district, Mueang Mae Hong Son District, Mae Hong Son Province, Thailand (ZMKU AM 01546) in (**A**) dorsal view, (**B**) ventral view, (**C**) lateral view, and (**D**) plantar view of the right foot. Paratype female (FMNH 97974) of *L. doriae* in preservative in (**E**) dorsal view, (**F**) ventral view, and (**G**) plantar view of the right foot. Arrows refer to diagnostic characters in toe webbing.

**Figure 13:**
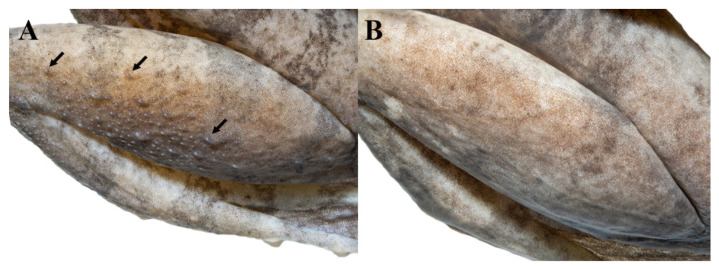
Comparison of dorsal surfaces of the shank showing tubercles (arrows) in adult males of (**A**) the holotype of *Limnonectes pseudodoriae* **sp. nov**. (ZMKU AM 01567), and (**B**) *L. doriae* (ZMKU AM 01546) from Mok Champae District, Mueang Mae Hong Son District, Mae Hong Son Province, Thailand.

*Limnonectes pseudodoriae* **sp. nov**. is morphologically most similar to *L. doriae* (Figure 12). The new species differs from *L. doriae* by having a smaller body size (Figure 12, Tables S2 and S3), with SVL of adult males 42.6–48.2 mm and adult females 36.0–44.1 mm (SVL of adult males SVL 41.4–55.0 mm and adult females 39.4–50.3 mm in *L. doriae*); dense, grained translucent spinules on broadened convex skin on the interorbital region (no spinules in *L. doriae*); more enlarged oval tubercles and slightly elongated tubercles on dorsum, not arranged in rows (fewer tubercles and arranged in rows in *L. doriae*); distinct, moderately dense, small homogenously-sized tubercles tipped with translucent spinules on dorsal surface of shank (Figure 13A) (less dense, smooth or indistinct, homogenously-sized tubercles tipped with translucent spinules in *L. doriae*, Figure 13B); chin, throat, chest, belly and ventral surfaces of forelimb and groin yellowish white (white in *L. doriae*); no dermal ridge along the outer side of Toe I (present in *L. doriae*); inner metatarsal tubercle approximately 35% the length of Toe I (approximately 40% in *L. doriae*); and toe webbing on the preaxial side of Toe III to the level of midway between distal and proximal subarticular tubercles and continuing as a fringe to base of tip (Figure 5E) (to proximal subarticular tubercle and continuing as a fringe to base of tip in *L. doriae*, Figure 12D,G).

Advertisement call characteristics are available for four closely related species: *L. dabanus*, *L. doriae*, *L. lauhachindai*, and *L. savan* [7,8,17], in this study The male advertisement call of *L. pseudodoriae* **sp. nov**. differs from all four of these species by having two undistinguished pulses per note (one-pulsed in *L. dabanus*, *L. doriae*, and *L. lauhachindai* [Type 1 call]; 31–37 pulses in *L. lauhachindai* [Type 2 call]; and 19–20 pulses in *L. savan*); 1–10 notes per call (one note in *L. dabanus* and *L. savan*); call duration of 124.56–1965.07 ms (57–76 ms in *L. savan*); note duration of 117.65–183.86 ms (60.88–113.15 ms and 72.56–77.62 ms in *L. doriae* [Type A and Type B calls, respectively], 10.29–34.50 ms and 1749.06–2148.03 ms in *L. lauhachindai* [Type 1 and Type 2 calls, respectively], 57–76 ms in *L. savan*); internote interval of 7.97–75.05 ms (182.50–972.41 ms in *L. lauhachindai* [Type 1 call]); note rate of 4.55–7.11 note per second (7.82–13.23 and 11.54–11.77 notes per second in *L. doriae* [Type A and Type B calls, respectively]); and dominant frequency of 0.52–1.03 kHz (1.12–2.33 and 2.15–2.24 kHz in *L. lauhachindai* [Type 1 and Type 2 calls, respectively]).

## 4. Discussion

The new species morphologically resembles *L. doriae*, but detailed comparisons of qualitative morphological characters, morphometrics, and statistical analyses revealed clear differences between them. These differences in morphology were corroborated by the finding that the new species represents a deeply divergent mtDNA lineage (both in genetic *p*-distances and in the molecular phylogeny) compared to its congeners. Last, the new species was readily differentiated from *L. doriae* in the temporal parameters of the advertisement calls. Therefore, the congruence in mtDNA, morphology, and advertisement calls supported the validity of *L. pseudodoriae* **sp. nov**. The phylogenetic analysis in this study revealed that the new species belongs to a clade containing *L. coffeatus*, *L. doriae*, *L. hascheanus*, *L. limborgi*, *L. macrognathus*, and *L. plicatellus*, but unfortunately, the exact sister taxon relationship of the new species was not resolved. These phylogenetic relationships were also unresolved in other studies (e.g., [17]), despite our addition here of the *ND3* gene to the more commonly used *16S* gene. Additional sequence data (nuclear and/or additional mitochondrial markers) are probably needed to better resolve these relationships.

Morphological characters alone often fail or are limited in their ability to recognize and delimit cryptic species of anurans (e.g., [52,53,54,55]). The incorporation of additional lines of evidence (e.g., molecular or bioacoustics) has been useful in uncovering cryptic diversity and clarifying taxonomic problems. In frogs, the advertisement call is usually species- specific and serves an important function in reproductive behavior [56,57]. As shown here with the new species and *L. doriae*, these data can greatly aid in delimiting sympatric or morphologically cryptic species (e.g., [8,50,52,58,59,60,61,62]).

In Thailand, *L. doriae* is patchily distributed in mountainous habitats from the northwestern region (Tak Province) to the southern region (Chumphon Province or probably through to Trang Province) of the country [14,15]. In this study, we extended the geographic range of *L. doriae* northward into Mae Hong Son Province. Some authors have suggested that the southern limits of the geographic range of *L. doriae* extend throughout southern Thailand and into Peninsular Malaysia [1,11,12,13]. *Limnonectes pseudodoriae* **sp. nov**. is currently known only from three islands in southern Thailand, Ko Pha-ngan and Ko Samui Islands on the Gulf of Thailand coast, and Ko Lanta Yai Island on the Andaman Sea coast. At those localities, we did not find *L. doriae*. The presence of *L. pseudodoriae* **sp. nov**. on the mainland, and the details of its geographic separation from *L. doriae*, remain unknown. It is possible that records of *L.* “*doriae*” from extreme southern Thailand and Peninsular Malaysia are actually *L. pseudodoriae* **sp. nov**., and that the two species are separated by the Khlong Mauri Fault south of the Isthmus of Kra [63,64], a known historical barrier to dispersal and stimulant for vicariant speciation in many organisms (e.g., [65,66,67]). Additional field sampling for *L. doriae* and *L. pseudodoriae* **sp. nov**., in southern Thailand, southern Myanmar, and Peninsular Malaysia, as well as re-evaluation of existing museum specimens, is needed to determine the actual geographic ranges of these two species. Moreover, additional field work is needed to understand the full breadth of the ecological requirements of *L. pseudodoriae* **sp. nov**. to effectively conserve the new species.

## 5. Conclusions

We described a new species of the fanged frog genus *Limnonectes* (Amphibia, Anura, Dicroglossidae) from southern Thailand, Ko Pha-ngan and Ko Samui Islands (the Gulf of Thailand coast) and Ko Lanta Yai Island (the Andaman Sea coast), using an integrative taxonomic approach based on multiple lines of evidence. *Limnonectes pseudodoriae* **sp. nov**. can be differentiated from the most morphologically similar species, *L. doriae*, and its congeners on the basis of mitochondrial DNA (*16S rRNA* and *ND3*), qualitative and quantitative morphology, and advertisement calls. However, the exact sister relationship of the new species remains unresolved, and additional sequence data may be required to resolve these relationships. *Limnonectes pseudodoriae* **sp. nov**. is known only from a relatively small geographic area in southern Thailand, where it inhabits mountain streams at mid to low elevations similar to many other fanged frogs. The new species deposits eggs in terrestrial nests near streams that consist of moist, shallow, clay holes covered with leaf litter, and the tadpoles develop in the moist clay of the nest, without free water or in only a small volume of fluid. Additional field sampling is needed to understand the extent of the geographic range and natural history of the new species.

## Figures and Tables

**Table 1:** Percentage uncorrected pairwise sequence divergences (*p*-distances) in the mitochondrial *16S* rRNA gene of *Limnonectes pseudodoriae* **sp. nov**. and related species.

*Limnonectes* Species	*pseudodoriae* sp. nov.	*doriae*	*limborgi*	*hascheanus*	*macrognathus*	*plicatellus*	*kohchangae*	*coffeatus*	*lauhachindai*	*gyldenstolpei*	*dabanus*	*savan*
*pseudodoriae* **sp. nov.** (*n* = 11)	0.6 (0.0–1.3)											
*doriae* (*n* = 17)	11.8 (10.6–13.8)	2.3 (0.0–5.9)										
*limborgi* (*n* = 3)	8.8 (8.6–9.4)	6.6 (6.0–8.1)	0.4 (0.0–0.6)									
*hascheanus* (*n* = 3)	10.3 (10.1–10.8)	7.6 (7.1–8.6)	7.6 (7.5–7.7)	0.0 (0.0)								
*macrognathus* (*n* = 3)	12.6 (11.9–13.3)	7.8 (7.2–9.7)	4.9 (4.7–5.3)	6.4 (6.2–6.7)	0.5 (0.0–0.8)							
*plicatellus* (*n* = 3)	12.8 (12.6–13.3)	8.7 (8.1–10.6)	8.7 (8.0–9.8)	9.7 (9.1–10.2)	9.7 (8.6–10.5)	3.8 (1.5–5.1)						
*kohchangae* (*n* = 3)	11.6 (10.8–13.1)	8.7 (7.4–10.4)	7.1 (6.8–7.7)	8.3 (8.0–8.6)	9.3 (8.7–10.4)	9.0 (8.4–10.2)	2.4 (0.1–3.7)					
*coffeatus* (*n* = 3)	14.9 (14.7–15.1)	11.7 (10.7–12.9)	10.5 (10.4–10.6)	10.6 (10.6)	12.2 (11.6–12.7)	10.7 (9.6–11.4)	10.6 (10.2–11.5)	0.0 (0.0)				
*lauhachindai* (*n* = 3)	12.6 (12.6)	11.4 (11.0–13.7)	12.3 (12.3)	13.9 (13.9)	11.7 (11.3–12.4)	12.2 (11.4–13.0)	10.6 (10.4–10.8)	13.5 (13.5)	0.0 (0.0)			
*gyldenstolpei* (*n* = 3)	16.6 (16.2–17.1)	14.7 (13.6–16.6)	12.7 (12.6–13.0)	12.8 (12.8)	15.3 (14.7–15.8)	14.9 (13.9–15.5)	13.8 (13.4–14.4)	16.4 (16.2–16.5)	7.2 (7.1–7.5)	1.0 (0.4–1.5)		
*dabanus* (*n* = 3)	15.9 (15.3–16.5)	13.4 (12.3–15.1)	12.3 (11.3–13.2)	12.0 (11.3–12.8)	14.1 (13.4–14.8)	13.7 (13.2–14.7)	12.3 (11.8–13.2)	15.2 (14.9–15.6)	6.6 (6.1–7.1)	8.5 (8.2–9.0)	0.3 (0.0–0.5)	
*savan* (*n* = 3)	17.0 (16.6–17.1)	13.1 (12.0–14.8)	11.9 (11.7–12.1)	12.2 (12.0–12.2)	14.1 (13.3–14.7)	13.7 (12.6–14.9)	13.4 (13.1–13.8)	15.6 (15.5–15.7)	6.7 (6.6–6.7)	8.3 (8.1–8.4)	6.6 (6.1–7.1)	0.2 (0.1–0.3)

**Table 2:** Percentage uncorrected pairwise sequence divergences (*p*-distances) in the mitochondrial *ND3* gene of *Limnonectes pseudodoriae* **sp. nov**. and related species.

*Limnonectes* Species	*pseudodoriae* sp. nov.	*doriae*	*limborgi*	*hascheanus*	*kohchangae*	*gyldenstolpei*	*dabanus*	*fragilis*	*fujiangensis*	*banaensis*	*jarujini*	*blythii*
*pseudodoriae* **sp. nov**. (*n* = 11)	0.9 (0.0–2.0)											
*doriae* (*n* = 15)	18.7 (17.4–19.3)	3.8 (0.0–8.8)										
*limborgi* (*n* = 3)	19.9 (19.6–20.0)	15.0 (14.2–15.8)	0.3 (0.0–0.5)									
*hascheanus* (*n* = 3)	20.2 (19.6–20.6)	16.7 (15.6–17.2)	15.9 (15.8–15.9)	0.3 (0.3–0.4)								
*kohchangae* (*n* = 1)	19.6 (19.1–19.9)	14.5 (14.3–15.2)	16.1 (16.1–16.2)	17.1 (17.1–17.2)	-							
*gyldenstolpei* (*n* = 1)	22.2 (22.2–22.3)	17.3 (16.3–18.5)	20.0 (20.0–20.1)	20.4 (20.3–20.5)	19.7 (19.7)	-						
*dabanus* (*n* = 1)	22.6 (22.5–22.6)	18.5 (18.1–18.9)	20.6 (20.4–20.7)	19.6 (19.6–19.7)	19.1 (19.1)	15.8 (15.8)	-					
*fragilis* (*n* = 1)	24.0 (23.9–24.1)	19.7 (19.4–20.3)	22.4 (22.4)	21.5 (21.5–21.6)	19.7 (19.7)	20.0 (20.0)	19.6 (19.6)	-				
*fujiangensis* (*n* = 1)	23.3 (23.1–23.5)	20.7 (20.3–21.4)	22.1 (22.0–22.2)	21.7 (21.6–21.8)	20.9 (20.9)	20.5 (20.5)	21.6 (21.6)	20.6 (20.6)	-			
*banaensis* (*n* = 1)	24.1 (23.9–24.2)	20.9 (20.7–21.3)	22.0 (21.8–22.2)	22.2 (22.1–22.3)	21.3 (21.3)	20.6 (20.6)	20.2 (20.2)	20.5 (20.5)	16.1 (16.1)	-		
*jarujini* (*n* = 1)	25.2 (24.9–25.3)	21.0 (20.7–21.8)	22.2 (22.1–22.3)	21.4 (21.4–21.5)	19.7 (19.7)	20.5 (20.5)	21.0 (21.0)	19.7 (19.7)	17.2 (17.2)	16.2 (16.2)	-	
*blythii* (*n* = 1)	23.5 (23.1–23.6)	20.5 (20.2–21.2)	22.7 (22.6–22.7)	21.5 (21.4–21.6)	20.7 (20.7)	21.4 (21.4)	21.2 (21.2)	20.7 (20.7)	22.4 (22.4)	21.9 (21.9)	21.5 (21.5)	-

**Table 3:** Factor loadings on the first three principal components (PC) of 16 morphological characters of males and females of *Limnonectes pseudodoriae* **sp. nov**. and *L. doriae*. Bold figures indicate high loadings.

Character	Male			Female		
	PC 1	PC 2	PC 3	PC 1	PC 2	PC 3
SVL_adj_	0.424	0.183	−0.121	**0.651**	−0.049	−0.190
HDL_adj_	−0.122	**0.776**	0.309	**0.933**	0.201	−0.112
HWL_adj_	−0.324	**0.875**	0.066	**0.947**	−0.046	0.151
SNT_adj_	−0.031	**0.716**	0.143	**0.917**	0.043	0.155
EYE_adj_	**0.704**	−0.075	0.017	**0.770**	−0.475	0.259
IOD_adj_	0.219	**0.699**	−0.003	**0.866**	−0.105	−0.102
IND_adj_	**0.778**	0.463	−0.107	**0.862**	−0.316	0.025
SHK_adj_	**0.858**	0.195	0.061	**0.969**	0.038	−0.007
TGH_adj_	**0.914**	0.048	0.180	**0.907**	0.243	−0.104
LAL_adj_	0.008	−0.273	**0.878**	**0.729**	0.336	−0.058
HND_adj_	**0.653**	−0.261	0.240	**0.725**	0.541	−0.290
FTL_adj_	**0.899**	0.166	0.270	**0.955**	0.113	−0.102
IML_adj_	**0.860**	0.198	−0.054	**0.939**	0.071	−0.077
IMW_adj_	0.433	**0.624**	−0.351	**0.788**	−0.199	0.439
TMP_adj_	**−0.773**	0.517	0.162	0.079	**0.865**	0.416
TMP/EYE	**−0.850**	0.431	0.133	−0.471	**0.855**	0.093
Eigenvalue	6.503	3.716	1.255	10.578	2.387	0.663
Percentage of variance	40.643	23.228	7.844	66.110	14.920	4.142
Cumulative proportion	40.643	63.871	71.715	66.110	81.030	85.172

**Table 4:** Statistical comparisons of males and females of *Limnonectes pseudodoriae* **sp. nov**. and *L. doriae*.

Characters	Males				Females			
	*pseudodoriae* sp. nov.	*doriae*	*t*	*p*	*pseudodoriae* sp. nov.	*doriae*	*t*	*p*
	*n* = 18	*n* = 16			*n* = 14	*n* =14		
SVL	45.2 ± 1.7 (42.6–48.2)	47.8 ± 3.7 (41.4–55.0)	2.513 ^a^	0.021 *	40.0 ± 2.8 (36.0–44.1)	45.00± 3.3 (39.4–50.3)	4.282	<0.001 *
HDL_adj_	21.56 ± 0.78 (19.91–22.61)	21.92 ± 1.27 (18.53–23.42)	185.000 ^b^	0.162	16.69 ± 0.33 (16.17–17.29)	18.38 ± 0.46 (17.71–19.12)	11.136	<0.001 *
HDW_adj_	21.61 ± 1.12 (18.91–22.93)	21.61 ± 1.02 (19.78–23.36)	0.019	0.985	15.91 ± 0.51 (15.10–16.73)	17.84 ± 0.47 (16.85–18.67)	196.000 ^b^	<0.001 *
SNT_adj_	8.23 ± 0.40 (7.27–8.91)	8.38 ± 0.30 (7.92–8.99)	1.211	0.235	6.52 ± 0.20 (6.16–6.85)	7.41 ± 0.24 (7.18–8.14)	196.000 ^b^	<0.001 *
EYE_adj_	4.70 ± 0.22 (4.28–5.09)	5.03 ± 0.28 (4.58–5.63)	3.770	0.001 *	4.34 ± 0.20 (3.98–4.70)	4.89 ± 0.31 (4.25–5.40)	5.641	<0.001 *
IOD_adj_	4.76 ± 0.42 (3.88–5.23)	5.11 ± 0.49 (4.42–6.23)	201.000 ^b^	0.051	3.34 ± 0.20 (3.00–3.62)	3.94 ± 0.25 (3.40–4.47)	7.088	<0.001 *
IND_adj_	3.76 ± 0.21 (3.49–4.24)	4.81 ± 0.32 (4.10–5.32)	11.433	<0.001 *	3.17 ± 0.28 (2.69–3.65)	4.32 ± 0.41 (3.65–5.17)	8.691	<0.001 *
SHK_adj_	22.72 ± 0.76 (21.13–24.02)	25.15 ± 1.23 (23.16–27.32)	7.026	<0.001 *	20.04 ± 1.02 (18.55–21.96)	23.86 ± 0.50 (23.28–25.12)	12.588 ^a^	<0.001 *
TGH_adj_	23.86 ± 0.49 (22.89–24.71)	26.03 ± 1.02 (24.40–28.16)	7.776 ^a^	<0.001 *	21.59 ± 0.82 (20.41–23.07)	24.82 ± 1.21 (22.22–26.51)	8.283	<0.001 *
LAL_adj_	9.87 ± 0.44 (9.06–10.59)	9.67 ± 0.60 (8.77–10.81)	107.500 ^b^	0.214	8.99 ± 0.29 (8.72–9.84)	9.40 ± 0.27 (9.07–9.90)	175.000 ^b^	<0.001 *
HND_adj_	11.18 ± 0.49 (10.30–12.06)	11.62 ± 0.44 (10.91–12.60)	2.748	0.010 *	9.87 ± 0.36 (9.29–10.52)	10.76 ± 0.63 (9.57–11.70)	4.543	<0.001 *
FTL_adj_	22.56 ± 0.45 (21.86–23.42)	24.87 ± 1.00 (23.03–25.91)	281.500 ^b^	<0.001 *	20.08 ± 0.73 (18.87–21.54)	23.96 ± 0.90 (22.78–25.93)	12.556	<0.001 *
IML_adj_	2.94 ± 0.17 (2.65–3.24)	3.69 ± 0.26 (3.08–4.01)	9.991	<0.001 *	2.44 ± 0.17 (2.24–2.74)	3.42 ± 0.24 (3.06–3.92)	12.651	<0.001 *
IMW_adj_	1.21 ± 0.15 (0.90–1.51)	1.45 ± 0.18 (1.18–1.76)	4.204	<0.001 *	1.04 ± 0.08 (0.90–1.21)	1.24 ± 0.11 (1.07–1.38)	5.516	<0.001 *
TMP_adj_	5.82 ± 0.67 (4.16–6.50)	5.06 ± 0.54 (4.18–6.14)	53.000 ^b^	0.002 *	3.44 ± 0.22 (3.09–3.88)	3.44 ± 0.30 (2.88–3.91)	0.058	0.058
TMP/EYE	1.24 ± 0.17 (0.82–1.43)	1.01 ± 0.13 (0.76–1.20)	39.500 ^b^	<0.001 *	0.79 ± 0.06 (0.72–0.94)	0.71 ± 0.09 (0.52–0.84)	−2.901	0.007 *

Data are given as mean and standard deviation, followed by range in parentheses. Key: ^a^ tested by Welch *t*-test, ^b^ tested by Mann–Whitney U test, * significance level at *p* < 0.05.

**Table 5:** Call parameters of *Limnonectes pseudodoriae* **sp. nov**. and *L. doriae.* Parameter values are given as mean ± SD and ranges in parentheses.

Call Parameters	*L. pseudodoriae* sp. nov. *n*_individual_ = 3	*L. doriae* *n*_ndividual_ = 1	
Call type	*n*_call_ = 180	Type A *n*_call_ = 6	Type B *n*_call_ = 3
Note description	two-pulsed note	non-pulsed note	
Call duration (ms)	335.66 ± 333.62 (124.56–1965.07)	403.08 ± 249.40 (151.28–820.60)	7253.86 ± 398.90 (7012.09–7714.28)
Intercall interval (s)	1.28 ± 1.52 (0.38–10.84)	108.22 ± 189.88 (0.41–451.06)	
Call rate (call/min)	37.65 ± 7.62 (32.46–46.39)	0.56	
Number of note (note)	1.98 ± 1.62 (1–10)	5.17 ± 3.43 (2–11)	84.67 ± 5.51 (81–91)
Note duration (ms)	139.94 ± 12.69 (117.65–183.86)	75.83 ± 21.13 (60.88–113.15)	75.58 ± 2.67 (72.56–77.62)
Internote interval (ms)	41.42 ± 11.94 (7.97–75.05)	11.05 ± 2.82 (7.54–14.76)	11.44 ± 3.55 (8.48–15.37)
Note rate (notes/s)	5.60 ± 0.47 (4.55–7.11)	11.32 ± 2.02 (7.82–13.23)	11.65 ± 0.11 (11.54–11.77)
Dominant frequency (kHz)	0.89 ± 0.13 (0.52–1.03)	0.92 ± 0.25 (0.60–1.12)	0.98 ± 0.25 (0.69–1.12)
Temperature (°C)	26.0–26.1	24.7	

## Data Availability

The data presented in this study are available on request from the corresponding author.

## Supplementary Materials

The following are available online at https://www.mdpi.com/2076-2615/11/2/566/s1, Data S1: Comparative specimens examined, Table S1: Specimens of *Limnonectes* used in (A) molecular and/or (B) morphological analyses in this study, Table S2: Morphological measurements (mm) of adult male specimens of *Limnonectes pseudodoriae* **sp. nov**. and *L. doriae*. Data are given as mean and standard deviation, followed by range in parentheses, Table S3: Morphological measurements (mm) of adult female specimens of *Limnonectes pseudodoriae* **sp. nov**. and *L. doriae*. Data are given as mean and standard deviation, followed by range in parentheses, and Table S4: Call parameters of three male paratypes of *Limnonectes pseudodoriae* **sp. nov**. Parameter values are given as mean ± SD and ranges in parentheses.

## References

[1] Frost D.R. (2020). Amphibian Species of the World: An Online Reference Version 6.0. http://research.amnh.org/vz/herpetology/amphibia/.

[2] Boulenger G.A. (1920). A monograph of the South Asian, Papuan, Melanesian and Australian frogs of the genus *Rana*. Rec. Indian Museum..

[3] Pope C.H. (1931). Notes on amphibians from Fukien, Hainan, and other parts of China. Bull. Am. Mus. Nat..

[4] Inger R.F. (1966). The systematic and zoogeography of the amphibia in Borneo. Fieldiana Zool..

[5] Emerson S.B. (2001). A macroevolutionary study of historical contingency in the fanged frogs of Southeast Asia. Biol. J. Linn. Soc..

[6] Lambertz M., Hartmann T., Walsh S., Geissler G., McLeod D.S. (2014). Anatomy, histology, and systematic implications of the head ornamentation in the males of four species of *Limnonectes* (Anura: Dicroglossidae). Zool. J. Linn. Soc..

[7] Rowley J.J.L., Le D.T.T., Hoang H.D., Altig R. (2014). The breeding behaviour advertisement call and tadpole of *Limnonectes dabanus* (Anura: Dicroglossidae). Zootaxa.

[8] Aowphol A., Rujirawan A., Taksintum W., Chuaynkern Y., Stuart B.L. (2015). A new caruncle-bearing *Limnonectes* (Anura: Dicroglossidae) from northeastern Thailand. Zootaxa.

[9] Emerson S.B. (1994). Testing pattern predictions of sexual selection: A frog example. Am. Nat..

[10] Emerson S.B., Inger R.F., Iskandar D. (2000). Molecular systematics and biogeography of the fanged frogs of Southeast Asia. Mol. Phylogenet. Evol..

[11] Berry P.Y. (1975). The Amphibian Fauna of Peninsular Malaysia.

[12] Taylor E.H. (1962). The amphibian fauna of Thailand. Univ. Kans. sci. bull..

[13] Chan-ard T.A. (2003). Photographic Guide to Amphibians in Thailand.

[14] Chuaynkern Y., Chuaynkern C. (2012). A checklist of amphibians in Thailand. J. Wildl. Thail..

[15] Niyomwan P., Srisom P., Pawangkhanant P. (2019). Amphibians of Thailand.

[16] Inger R.F., Stuart B.L. (2010). Systematics of *Limnonectes* (*Taylorana*) Dubois. Curr. Herpetol..

[17] Phimmachak S., Richards S.J., Sivongxay N., Saeteun S., Chuaynkern Y., Makchai S., Som H.E., Stuart B.L. (2019). A caruncle-bearing fanged frog (*Limnonectes*, Dicroglossidae) from Laos and Thailand. Zookeys.

[18] Phimmachak S., Sivongxay N., Saeteun S., Yodthong S., Rujirawan A., Neang T., Aowphol A., Stuart B.L. (2018). A new *Limnonectes* (Anura: Dicroglossidae) from southern Laos. Zootaxa.

[19] Simmons J.E. (2015). Herpetological Collecting and Collections Management.

[20] Chen L., Murphy R.W., Lathrop A., Ngo A., Orlov N.L., Ho C.T., Somorjai I.L.M. (2005). Taxonomic chaos in Asian ranid frogs: An initial phylogenetic resolution. Herpetol. J..

[21] Palumbi S.R., Hills D.M., Moritz C., Moble B.K. (1996). Nucleic acids II: The polymerase chain reaction. Molecular Systematics.

[22] Stuart B.L., Chuaynkern Y., Chan-ard T., Inger R.F. (2006). Three new species of frogs and a new tadpole from eastern Thailand. Fieldiana Zool..

[23] Inger R.F., Stuart B.L., Iskandar D.T. (2009). Systematics of a widespread Southeast Asian frog, *Rana chalconota* (Amphibia: Anura: Ranidae). Zool. J. Linn. Soc..

[24] Evans B.J., Brown R.M., McGuire J.A., Supriatna J., Andayani N., Diesmos A., Iskandar D., Melnick D.J., Cannatella D.C. (2003). Phylogenetics of fanged frogs: Testing biogeographical hypotheses at the interface of the Asian and Australian faunal zones. Syst. Biol..

[25] Pyron R.A., Wiens J.J. (2011). A large-scale phylogeny of amphibia including over 2800 specie, and a revised classification of extant frogs, salamanders, and caecilians. Mol. Phylogenet. Evol..

[26] Lanfear R., Frandsen P.B., Wright A.M., Senfeld T., Calcott B. (2016). PartitionFinder 2: New methods for selecting partitioned models of evolution for molecular and morphological phylogenetic analyses. Mol. Biol. Evol..

[27] Miller M.A., Pfeiffer W., Schwartz T. (2010). Creating the CIPRES science gateway for inference of large phylogenetic trees. 2010 Gateway Computing Environments Workshop (GCE), New Orleans, Louisiana, USA, 14 November 2010.

[28] Ronquist F., Teslenko M., Van Der Mark P., Ayres D.L., Darling A., Höhna S., Larget B., Liu L., Suchard M.A., Huelsenbeck J.P. (2012). MrBayes 3.2: Efficient Bayesian phylogenetic inference and model choice across a large model space. Syst. Biol..

[29] Rambaut A., Drummond A.J., Xie D., Baele G., Suchard M.A. (2018). Posterior summarisation in Bayesian phylogenetics using Tracer 1.7. Syst. Biol..

[30] Nguyen L.T., Schmidt H.A., von Haeseler A., Minh B.Q. (2015). IQ-TREE: A fast and effective stochastic algorithm for estimating maximum-likelihood phylogenies. Mol. Biol. Evol..

[31] Trifinopoulos J., Nguyen L.T., von Haeseler A., Minh B.Q. (2016). W-IQ-TREE: A fast online phylogenetic tool for maximum likelihood analysis. Nucleic Acids Res..

[32] Hoang D.T., Chernomor O., von Haeseler A., Minh B.Q., Vinh L.S. (2018). UFBoot2: Improving the ultrafast bootstrap approximation. Mol. Biol. Evol..

[33] Minh Q., Nguyen M.A.T., von Haeseler A. (2013). Ultrafast approximation for phylogenetic bootstrap. Mol. Biol. Evol..

[34] Kumar S., Stecher G., Li M., Knyaz C., Tamura K. (2018). MEGA X: Molecular Evolutionary Genetics Analysis across computing platforms. Mol. Biol. Evol..

[35] Savage J.M., Heyer W.R. (1967). Variation and distribution in the tree-frog genus *Phyllomedusa* in Costa Rica, Central America. Beiträge Neotropischen Fauna.

[36] Sclater W.L. (1892). On some specimens of frogs in the Indian Museum, Calcutta, with descriptions of several new species. Proc. Zool. Soc. Lond..

[37] Stoliczka F. (1870). Observations on some Indian and Malayan Amphibia and Reptilia. J. Asiat. Soc. Benga..

[38] Boulenger G.A. (1887). An account of the reptiles and batrachians obtained in Tenasserim by M. L. Fea of the Genoa Civic Museum. Ann. Mus. Civ. Stor. Nat. Genova.

[39] Smith M.A. (1922). The frogs allied to *Rana doriae*. Nat. Hist. Bull. Siam. Soc..

[40] Smith M.A. (1922). The frogs allied to *Rana doriae*. Addendum. Nat. Hist. Bull. Siam. Soc..

[41] Thorpe R.S. (1975). Quantitative handling of characters useful in snake systematics with particular reference to intraspecific variation in the Ringed Snakes *Natrix natrix* (L.). Biol. J. Linn. Soc..

[42] Lleonart J., Salat J., Torres G.J. (2000). Removing allometric effects of body size in morphological analysis. J. Theor. Biol..

[43] Sheridan J.A., Stuart B.L. (2018). Hidden species diversity in *Sylvirana nigrovittata* (Amphibia: Ranidae) highlights the importance of taxonomic revisions in biodiversity conservation. PLoS ONE.

[44] Lê S., Josse J., Husson F. (2008). FactoMineR: An R Package for Multivariate Analysis. J. Stat. Softw..

[45] Husson F., Le S., Pagès J. (2017). Exploratory Multivariate Analysis by Example Using R.

[46] R Development Core Team R: A Language and Environment for Statistical Computing. http://www.r-project.org.

[47] Sueur J., Aubin T., Simonis C. (2008). Seewave: A free modular tool for sound analysis and synthesis. Bioacoustics.

[48] Cocroft R.B., Ryan M.J. (1995). Patterns of advertisement call evolution in toads and chorus frogs. Anim. Behav..

[49] Thomas A., Suyesh R., Biju S.D., Bee M.A. (2014). Vocal behavior of the elusive purple frog of India (*Nasikabatrachus sahyadrensis*), a fossorial species endemic to the Western Ghats. PLoS ONE.

[50] Köhler J., Jansen M., Rodríguez A., Kok P.J.R., Toledo L.F., Emmrich M., Glaw F., Haddad C.F.B., Rödel M.O., Vences M. (2017). The use of bioacoustics in anuran taxonomy: Theory, terminology, methods and recommendations for best practice. Zootaxa.

[51] Emmrich M., Vences M., Ernst R., Köhler J., Barej M.F., Glaw F., Jansen M., Rödel M. (2020). A guild classification system proposed for anuran advertisement calls. Zoosyst. Evol..

[52] Kuramoto M., Joshy S.H., Kurabayashi A., Sumida M. (2007). The genus *Fejervarya* (Anura: Ranidae) in Central Western Ghats, India, with descriptions of four new cryptic species. Curr. Herpetol..

[53] Vences M., Köhler J., Crottini A., Glaw F. (2010). High mitochondrial sequence divergence meets morphological and bioacoustic conservatism: *Boophis quasiboehmei* sp. n., a new cryptic treefrog species from south-eastern Madagascar. Bonn. Zool. Bull..

[54] Rowley J.J.L., Tran D.T.A., Frankham G.J., Dekker A.H., Le D.T.T., Nguyen T.Q., Dau V.Q., Hoang H.D. (2015). Undiagnosed cryptic diversity in small, microendemic frogs (*Leptolalax*) from the Central Highlands of Vietnam. PLoS ONE.

[55] Dufresnes C., Mazepa G., Rodrigues N., Brelsford A., Litvinchuk S.N., Sermier R., Lavanchy G., Betto-Colliard C., Blaser O., Borzée A. (2018). Genomic evidence for cryptic speciation in tree frogs from the Apennine Peninsula, with description of *Hyla perrini* sp.. nov. Front. Ecol. Evol..

[56] Well K.D. (1977). The social behavior of anuran amphibians. Anim. Behav..

[57] Duellman W.E., Trueb L. (1994). Biology of Amphibians.

[58] Schneider H., Sinsch U., Heatwole H., Tyler M.J. (2007). Contributions of bioacoustics to the taxonomy of the Anura. Amphibian Biology.

[59] Rowley J.J.L., Stuart B.L., Thy N., Emmett D.A. (2010). A new species of *Leptolalax* (Anura: Megophryidae) from northeastern Cambodia. Zootaxa.

[60] Rujirawan A., Stuart B.L., Aowphol A. (2013). A new tree frog in the genus *Polypedates* (Anura: Rhacophoridae) from southern Thailand. Zootaxa.

[61] Liu Q.C., Wang T.L., Zhai X.F., Wang J.C. (2018). Call characteristics of two sympatric and morphologically similar tree frog species, *Polypedates megacephalus* and *Polypedates mutus* (Anura: Rhacophoridae), from Hainan, China. Asian Herpetol. Res..

[62] Stuart B.L., Som H.E., Neang T., Hoang H.D., Le D.T.T., Dau V.Q., Potter K., Rowley J.J.L. (2020). Integrative taxonomic analysis reveals a new species of *Leptobrachium* (Anura: Megophryidae) from north-eastern Cambodia and central Vietnam. J. Nat. Hist..

[63] Grismer L.L., Grismer J.L., Wood Jr P.L., Ngo V.T., Neang T., Chan K.O. (2011). Herpetology on the fringes of the sunda shelf: A discussion of discovery, taxonomy, and biogeography. Bonn. Zool. Monogr..

[64] Parnell J. (2013). The biogeography of the Isthmus of Kra region: A review. Nord. J. Bot..

[65] de Bruyn M., Nugroho E., Hossain M.M., Wilson J.C., Mather P.B. (2005). Phylogeographic evidence for the existence of an ancient biogeographic barrier: The Isthmus of Kra Seaway. Heredity.

[66] Bohlena J., Dvořáka T., Šlechtaa V., Šlechtováa V. (2020). Sea water shaping the freshwater biota: Hidden diversity and biogeographic history in the *Paracanthocobitis zonalternans* species complex (Teleostei: Nemacheilidae) in western Southeast Asia. Mol. Phylogenet. Evol..

[67] Bohlena J., Dvořáka T., Šlechtaa V., Šlechtováa V. (2020). Resolving an unnoticed diversity within the *Schistura robertsi* species complex (Teleostei: Nemacheilidae) using molecules and morphology. Mol. Phylogenet. Evol..

