# Supplementary material for: A New Limnonectes (Anura: Dicroglossidae) from Southern Thailand
Source: Animals (Basel). 2021 Feb 22;11(2):566. doi: 10.3390/ani11020566 (PMC7926908; doi:10.3390/ani11020566)
Supplement: Supplementary file 1 [file animals-11-00566-s001.zip › animals-1097152/animals-1097152-supplementary/Supplement_Data S1.docx]

**Data S1.** Comparative specimens examined.

*Limnonectes doriae*: **Myanmar** (“Burma”): Tenasserim: adult female FMNH 97974 (paratype); Tenasserim, Mooleyit (“Molleyil”): adult male CNHM 74155. **Thailand**: Mae Hong Son Province, Mueang Mae Hong Son District, Mok Champae Sub-district (19°29.470’N, 97°57.571’E): adult males, ZMKU AM 01546−47, ZMKU AM 01549, ZMKU AM 01552, adult females, ZMKU AM 01529, ZMKU AM 01548, ZMKU AM 01550−51; Tak Province, Mae Sot District, Mahawan Sub-district (16°34.210’N, 98°41.714’E): adult male, ZMKU AM 01527; Prachuap Khiri Khan Province, Thap Sakae District, Huai Yang Sub-district (11°37.694’N, 99°36.583’E): adult female ZMKU AM 01526; Chumphon Province, Sawi District, Thung Raya Sub-district (10°15.173’N, 98°56.763’E): adult male, ZMKU AM 01528; Ranong Province, Mueang Ranong District, Ngao Sub-district (9°51.410’N, 98°37.612’E): adult male, ZMKU AM 01523, adult females, ZMKU AM 01530–31, ZMKU AM 01539; Phang-nga Province, Kura Buri District, Moo Ko Surin National Park, Ko Surin Nuea (9°26.602’N, 97°52.303’E): adult males, ZMKU AM 01534–35, ZMKU AM 01538, ZMKU AM 01540, ZMKU AM 01542−44, adult females, ZMKU AM 015436–37, ZMKU AM 015441; Thai Mueang District, Thai Mueang Sub-district (8°28.018’N, 98°16.471’E): adult male, ZMKU AM 01524, adult females, ZMKU AM 0154332−33.

*Limnonectes kohchangae*: **Thailand**: Trat Province, Ko Chang District, Ko Chang Island (topotypes) (12°2.678’N, 102°18.276’E): adult female, ZMKU AM 00063, adult male, ZMKU AM 00066, (12° 3.892’ N, 102° 18.787’E): adult males, ZMKU AM 01155–56, 58, adult female, ZMKU AM 01157.
