# Supplementary material for: A New Limnonectes (Anura: Dicroglossidae) from Southern Thailand
Source: Animals (Basel). 2021 Feb 22;11(2):566. doi: 10.3390/ani11020566 (PMC7926908; doi:10.3390/ani11020566)
Supplement: Supplementary file 1 [file animals-11-00566-s001.zip › animals-1097152/animals-1097152-supplementary/Supplement_Table S1_with references.docx]

**Table S1.** Specimens of *Limnonectes* used in (A) molecular and/or (B) morphological analyses in this study.

| **Species** | **Locality** | **Locality No.** | **Museum No.** | **Type of Analyses** | **GenBank Accession No.** | | **Reference** |
| --- | --- | --- | --- | --- | --- | --- | --- |
|  |  |  |  |  | ***16S*** | ***ND3*** |  |
| *Fejervarya limnocharis* | China, Yancheng | - | Not available | A | NC_005055 | NC_005055 | [1] |
| *Quasipaa*  *spinosa* | China, Zhejiang Prov., Jinhua | - | Not available | A | NC_013270 | NC_013270 | [2] |
| *Limnonectes banaensis* | China, Yunnan Prov., Simao Dist. | - | ZNAC 21021 | A | AY899242 | AY899242 | [3] |
| *L. blythii* | Thailand, Prachuap Khiri Khan Prov., Huay Yang National Park | - | FMNH 268261 | A | GU934328 | GU934371 | [4] |
| *L. coffeatus* | Laos, Champasak Prov., Pakxong Dist. | - | FMNH 258440 | A | KY768794 | - | [5] |
| *L. coffeatus* | Laos, Champasak Prov., Pakxong Dist. | - | FMNH 258441 | A | KY768795 | - | [5] |
| *L. coffeatus* | Laos, Champasak Prov., Pakxong Dist. | - | NCSM 77785 | A | KY768796 | - | [5] |
| *L. dabanus* | Vietnam, Dac Lac Prov., Yok Don | - | ROM 22081 | A | AF206496 | - | [6] |
| *L. dabanus* | Cambodia, Mondolkiri Prov., Prichrada Dist., Phnom Nam Lyr Wildlife Sanctuary | - | FMNH 261937 | A | GU934329 | GU934372 | [4] |
| *L. dabanus* | Cambodia, Mondolkiri Prov., Pichrada Dist. | - | FMNH 261925 | A | MK688563 | - | [7] |
| *L. doriae* | Myanmar, Pegu State, Bago Yoma | 15 | CAS 208425 | A | GU934330 | GU934373 | [4] |
| *L. doriae* | Myanmar, Rakhine State, Rakhine Yoma | 16 | CAS 211632 | A | MK688573 | - | [7] |
| *L. doriae* | Myanmar, Tanintharyi Div., Kawthaung Dist. | 17 | CAS 229581 | A | MK688574 | - | [7] |
| *L. doriae* | Myanmar, Tenasserim | - | CNHM 74155 | B | - | - | This study |
| *L. doriae* (paratype) | Myanmar, Tenasserim | 14 | FMNH 97974 | B | - | - | This study |
| *L. doriae* | Thailand, Chumphon Prov., Phato Dist., Pak Song Sub-dist. | 9 | ZMKU AM 01521 | A | MW567806 | MW574596 | This study |
| *L. doriae* | Thailand, Chumphon Prov., Phato Dist., Pak Song Sub-dist. | 9 | ZMKU AM 01522 | A | MW567807 | MW574597 | This study |
| *L. doriae* | Thailand, Ranong Prov., Mueang Ranong Dist., Ngao Sub-dist. | 10 | ZMKU AM 01523 | A, B | MW567808 | MW574598 | This study |
| *L. doriae* | Thailand, Phang-nga Prov., Thai Mueang Dist., Thai Mueang Sub-dist. | 12 | ZMKU AM 01524 | A, B | MW567809 | MW574599 | This study |
| *L. doriae* | Thailand, Phang-nga Prov., Thai Mueang Dist., Thai Mueang Sub-dist. | 12 | ZMKU AM 01525 | A | MW567810 | MW574600 | This study |
| *L. doriae* | Thailand, Prachuap Khiri Khan Prov., Thap Sakae Dist., Huai Yang Sub-dist. | 7 | ZMKU AM 01526 | A, B | MW567811 | MW574601 | This study |
| *L. doriae* | Thailand, Tak Prov., Mae Sot Dist., Mahawan Sub-dist. | 6 | ZMKU AM 01527 | A, B | MW567812 | MW574602 | This study |
| *L. doriae* | Thailand, Chumphon Prov., Sawi Dist., Thung Raya Sub-dist., | 8 | ZMKU AM 01528 | A, B | MW567813 | MW574603 | This study |
| *L. doriae* | Thailand, Mae Hong Son Prov., Mueang Mae Hong Son Dist., Mok Champae Sub-dist. | 4 | ZMKU AM 01529 | B | - | - | This study |
| *L. doriae* | Thailand, Ranong Prov., Mueang Ranong Dist., Ngao Sub-dist. | 10 | ZMKU AM 01530 | A, B | MW567814 | MW574604 | This study |
| *L. doriae* | Thailand, Ranong Prov., Mueang Ranong Dist., Ngao Sub-dist. | 10 | ZMKU AM 01531 | B | - | - | This study |
| *L. doriae* | Thailand, Phang-nga Prov., Thai Mueang Dist., Thai Mueang Sub-dist. | 12 | ZMKU AM 01532 | B | - | - | This study |
| *L. doriae* | Thailand, Phang-nga Prov., Thai Mueang Dist., Thai Mueang Sub-dist. | 12 | ZMKU AM 01533 | B | - | - | This study |
| *L. doriae* | Thailand, Phang-nga Prov., Mu Ko Surin National Park, Ko Surin Nuea | 11 | ZMKU AM 01534 | A, B | MW567815 | MW574605 | This study |
| *L. doriae* | Thailand, Phang-nga Prov., Mu Ko Surin National Park, Ko Surin Nuea | 11 | ZMKU AM 01535 | B | - | - | This study |
| *L. doriae* | Thailand, Phang-nga Prov., Mu Ko Surin National Park, Ko Surin Nuea | 11 | ZMKU AM 01536 | B | - | - | This study |
| *L. doriae* | Thailand, Phang-nga Prov., Mu Ko Surin National Park, Ko Surin Nuea | 11 | ZMKU AM 01537 | B | - | - | This study |
| *L. doriae* | Thailand, Phang-nga Prov., Mu Ko Surin National Park, Ko Surin Nuea | 11 | ZMKU AM 01538 | B | - | - | This study |
| *L. doriae* | Thailand, Ranong Prov., Mueang Ranong Dist., Ngao Sub-dist. | 10 | ZMKU AM 01539 | B | - | - | This study |
| *L. doriae* | Thailand, Phang-nga Prov., Mu Ko Surin National Park, Ko Surin Nuea | 11 | ZMKU AM 01540 | B | - | - | This study |
| *L. doriae* | Thailand, Phang-nga Prov., Mu Ko Surin National Park, Ko Surin Nuea | 11 | ZMKU AM 01541 | B | - | - | This study |
| *L. doriae* | Thailand, Phang-nga Prov., Mu Ko Surin National Park, Ko Surin Nuea | 11 | ZMKU AM 01542 | B | - | - | This study |
| *L. doriae* | Thailand, Phang-nga Prov., Mu Ko Surin National Park, Ko Surin Nuea | 11 | ZMKU AM 01543 | A, B | MW567816 | MW574606 | This study |
| *L. doriae* | Thailand, Phang-nga Prov., Mu Ko Surin National Park, Ko Surin Nuea | 11 | ZMKU AM 01544 | B | - | - | This study |
| *L. doriae* | Thailand, Tak Prov., Tha Song Yang Dist., Mae Song Sub-dist. | 5 | ZMKU AM 01545 | A | MW567817 | MW574607 | This study |
| *L. doriae* | Thailand, Mae Hong Son Prov., Mueang Mae Hong Son Dist., Mok Champae Sub-dist. | 4 | ZMKU AM 01546 | B | - | - | This study |
| *L. doriae* | Thailand, Mae Hong Son Prov., Mueang Mae Hong Son Dist., Mok Champae Sub-dist. | 4 | ZMKU AM 01547 | A, B | MW567818 | MW574608 | This study |
| *L. doriae* | Thailand, Mae Hong Son Prov., Mueang Mae Hong Son Dist., Mok Champae Sub-dist. | 4 | ZMKU AM 01548 | B | - | - | This study |
| *L. doriae* | Thailand, Mae Hong Son Prov., Mueang Mae Hong Son Dist., Mok Champae Sub-dist. | 4 | ZMKU AM 01549 | B | - | - | This study |
| *L. doriae* | Thailand, Mae Hong Son Prov., Mueang Mae Hong Son Dist., Mok Champae Sub-dist. | 4 | ZMKU AM 01550 | B | - | - | This study |
| *L. doriae* | Thailand, Mae Hong Son Prov., Mueang Mae Hong Son Dist., Mok Champae Sub-dist. | 4 | ZMKU AM 01551 | B | - | - | This study |
| *L. doriae* | Thailand, Mae Hong Son Prov., Mueang Mae Hong Son Dist., Mok Champae Sub-dist. | 4 | ZMKU AM 01552 | A, B | MW567819 | MW574609 | This study |
| *L. fragilis* | Not available | - | ZNAC 11006 | A | - | - | GenBank submission |
| *L. fujianensis* | China, Fujian | - | Not available | A | - | - | GenBank submission |
| *L. glydenstolpei* | Thailand, Mueang Sa Kaeo Dist., Sa Kaeo Prov., Pang Si Da National Park | - | FMNH 266203 | A | GU934331 | GU934374 | [4] |
| *L. glydenstolpei* | Cambodia, Siem Reap Prov., Bante Sre Dist. | - | FMNH 257333 | A | MK688580 | - | [7] |
| *L. glydenstolpei* | Cambodia, Kampong Speu Prov., Aural Dist. | - | NCSM 79549 | A | MK688584 | - | [7] |
| *L. hascheanus* | Malaysia, Penang | - | LSUHC 6777 | A | GU934349 | GU934393 | [4] |
| *L. hascheanus* | Malaysia, Penang | - | LSUHC 6783 | A | GU934350 | GU934394 | [4] |
| *L. hascheanus* | Malaysia, Penang | - | LSUHC 8719 | A | GU934351 | GU934395 | [4] |
| *L. kadarsani* | Indonesia, Lombok Island | - | LSUMZ 81722 | A | AY313693 | - | [8] |
| *L. khasianus* | Thailand, Narathiwat Prov., Bala | - | KUHE 23158 | A | AB981414 | - | [9] |
| *L. kohchangae* | Cambodia, Kampong Speu Prov., Phnom Sruoch Dist., Kirirom National Park | - | FMNH 263210 | A | GU934332 | GU934375 | [4] |
| *L. kohchangae* | Thailand, Trat Prov., Ko Chang Dist. | - | ZMKU AM 01155 | A | KY768804 | - | [5] |
| *L. kohchangae* | Thailand, Trat Prov., Ko Chang Dist. | - | ZMKU AM 01157 | A | KY768805 | - | [5] |
| *L. jarujini* | Thailand, Prachuap Khiri Khan Prov., Huay Yang National Park | - | FMNH 268435 | A | GU 934333 | GU 934376 | [4] |
| *L. lauhachindai* | Thailand, Ubon Ratchathani Prov., Sirindhorn Dist. | - | NCSM 80222 | A | KP939072 | - | [10] |
| *L. lauhachindai* | Thailand, Ubon Ratchathani Prov., Sirindhorn Dist. | - | ZMKU AM 01104 | A | KP939073 | - | [10] |
| *L. lauhachindai* | Thailand, Ubon Ratchathani Prov., Sirindhorn Dist. | - | ZMKU AM 01105 | A | KP939074 | - | [10] |
| *L. leporinus* | Indonesia, Borneo Island, Kalimantan Timor Prov. | - | A167165 | A | AY313691 | - | [8] |
| *L. leytensis* | Philippines, Leyte Island | - | USNM222545 | A | JX911319 | - | [11] |
| *L. limborgi* | Myanmar, Kachin State, Mohnyin Township, Non-Yinkha Reserve | - | CAS 228513 | A | GU934353 | GU934397 | [4] |
| *L. limborgi* | Myanmar, Kachin State, Mohnyin Township, Non-Yinkha Reserve | - | CAS 228514 | A | GU934354 | GU934398 | [4] |
| *L. limborgi* | Myanmar, Sagaing Div., Homalin Township, Pidaung Wildlife Sanctuary | - | CAS 232167 | A | GU934359 | GU934403 | [4] |
| *L. macrognathus* | Thailand , Ranong Prov. | - | KUHE 23923 | A | AB971138 | - | [12] |
| *L. macrognathus* | Thailand, Surat Thani Prov., Kaeng Krung | - | FMNH 268503 | A | MK688588 | - | [7] |
| *L. macrognathus* | Thailand, Surat Thani Prov., Kaeng Krung | - | FMNH 268505 | A | MK688589 | - | [7] |
| *L. malesianus* | Malaysia, Sarawak Prov., Borneo Island | - | Not available | A | AY313692 | - | [8] |
| *L. microdiscus* | Indonesia, Java Island, Sakabumi | - | LSU81739 | A | AY313688 | - | [8] |
| *L. plicatellus* | Malaysia, Selangor Dist., Kepong, FRIM | - | LSUHC 4001 | A | KJ720983 | - | [13] |
| *L. plicatellus* | Malaysia, Selangor Dist., Gombak Swamp | - | LSUHC 6582 | A | KJ720982 | - | [13] |
| *L. plicatellus* | Malaysia, Pulau Pinang, Empangan Air Hitam | - | LSUHC 6710 | A | KJ720981 | - | [13] |
| *L. poilani* | Vietnam, Quang Nam Prov., Tre My, Tre Tep Commune | - | AMNH A163717 | A | DQ283378 | - | [14] |
| *L. pseudodoriae*  **sp. nov** | Thailand, Krabi Prov., Ko Lanta Dist., Ko Lanta Yai Island | 3 | ZMKU AM 01553 | A, B | MW567820 | MW574610 | This study |
| *L. pseudodoriae*  **sp. nov** | Thailand, Krabi Prov., Ko Lanta Dist., Ko Lanta Yai Island | 3 | ZMKU AM 01555 | A, B | MW567821 | MW574611 | This study |
| *L. pseudodoriae*  **sp. nov** | Thailand, Krabi Prov., Ko Lanta Dist., Ko Lanta Yai Island | 3 | ZMKU AM 01558 | B | - | - | This study |
| *L. pseudodoriae*  **sp. nov** | Thailand, Krabi Prov., Ko Lanta Dist., Ko Lanta Yai Island | 3 | ZMKU AM 01559 | B | - | - | This study |
|  |  |  |  |  |  |  |  |
| *L. pseudodoriae*  **sp. nov** | Thailand, Krabi Prov., Ko Lanta Dist., Ko Lanta Yai Island | 3 | ZMKU AM 01560 | B | - | - | This study |
| *L. pseudodoriae*  **sp. nov** | Thailand, Krabi Prov., Ko Lanta Dist., Ko Lanta Yai Island | 3 | ZMKU AM 01561 | B | - | - | This study |
| *L. pseudodoriae*  **sp. nov** | Thailand, Krabi Prov., Ko Lanta Dist., Ko Lanta Yai Island | 3 | ZMKU AM 01562 | B | - | - | This study |
| *L. pseudodoriae*  **sp. nov** | Thailand, Krabi Prov., Ko Lanta Dist., Ko Lanta Yai Island | 3 | ZMKU AM 01563 | A, B | MW567822 | MW574612 | This study |
| *L. pseudodoriae*  **sp. nov** | Thailand, Surat Thani Prov., Ko Phang-ngan Dist., Ko Pha-ngan Island | 1 | ZMKU AM 01565 | A, B | MW56723 | MW574613 | This study |
| *L. pseudodoriae*  **sp. nov** | Thailand, Surat Thani Prov., Ko Phang-ngan Dist., Ko Pha-ngan Island | 1 | ZMKU AM 01566 | B | - | - | This study |
| *L. pseudodoriae*  **sp. nov** | Thailand, Surat Thani Prov., Ko Phang-ngan Dist., Ko Pha-ngan Island | 1 | ZMKU AM 01567 | A, B | MW567824 | MW574614 | This study |
| *L. pseudodoriae*  **sp. nov** | Thailand, Surat Thani Prov., Ko Phang-ngan Dist., Ko Pha-ngan Island | 1 | ZMKU AM 01568 | B | - | - | This study |
| *L. pseudodoriae*  **sp. nov** | Thailand, Surat Thani Prov., Ko Phang-ngan Dist., Ko Pha-ngan Island | 1 | ZMKU AM 01569 | B | - | - | This study |
| *L. pseudodoriae*  **sp. nov** | Thailand, Surat Thani Prov., Ko Phang-ngan Dist., Ko Pha-ngan Island | 1 | ZMKU AM 01570 | B | - | - | This study |
| *L. pseudodoriae*  **sp. nov** | Thailand, Surat Thani Prov., Ko Phang-ngan Dist., Ko Pha-ngan Island | 1 | ZMKU AM 01571 | B | - | - | This study |
| *L. pseudodoriae*  **sp. nov** | Thailand, Surat Thani Prov., Ko Phang-ngan Dist., Ko Pha-ngan Island | 1 | ZMKU AM 01572 | B | - | - | This study |
| *L. pseudodoriae*  **sp. nov** | Thailand, Surat Thani Prov., Ko Phang-ngan Dist., Ko Pha-ngan Island | 1 | ZMKU AM 01573 | B | - | - | This study |
| *L. pseudodoriae*  **sp. nov** | Thailand, Surat Thani Prov., Ko Phang-ngan Dist., Ko Pha-ngan Island | 1 | ZMKU AM 01574 | B | - | - | This study |
| *L. pseudodoriae*  **sp. nov** | Thailand, Surat Thani Prov., Ko Phang-ngan Dist., Ko Pha-ngan Island | 1 | ZMKU AM 01575 | B | - | - | This study |
| *L. pseudodoriae*  **sp. nov** | Thailand, Surat Thani Prov., Ko Phang-ngan Dist., Ko Pha-ngan Island | 1 | ZMKU AM 01576 | B | - | - | This study |
| *L. pseudodoriae*  **sp. nov** | Thailand, Surat Thani Prov., Ko Phang-ngan Dist., Ko Pha-ngan Island | 1 | ZMKU AM 01577 | A, B | MW567825 | MW574615 | This study |
| *L. pseudodoriae*  **sp. nov** | Thailand, Surat Thani Prov., Ko Phang-ngan Dist., Ko Pha-ngan Island | 1 | ZMKU AM 01578 | B | - | - | This study |
| *L. pseudodoriae*  **sp. nov** | Thailand, Surat Thani Prov., Ko Phang-ngan Dist., Ko Pha-ngan Island | 1 | ZMKU AM 01579 | A, B | MW567826 | MW574616 | This study |
| *L. pseudodoriae*  **sp. nov** | Thailand, Surat Thani Prov., Ko Phang-ngan Dist., Ko Pha-ngan Island | 1 | ZMKU AM 01580 | A, B | MW567827 | MW574617 | This study |
| *L. pseudodoriae*  **sp. nov** | Thailand, Surat Thani Prov., Ko Phang-ngan Dist., Ko Pha-ngan Island | 1 | ZMKU AM 01581 | B | - | - | This study |
| *L. pseudodoriae*  **sp. nov** | Thailand, Surat Thani Prov., Ko Samui Dist., Ko Samui Island | 2 | ZMKU AM 01582 | A, B | MW567828 | MW574618 | This study |
| *L. pseudodoriae*  **sp. nov** | Thailand, Surat Thani Prov., Ko Samui Dist., Ko Samui Island | 2 | ZMKU AM 01583 | A, B | MW567829 | MW574619 | This study |
| *L. pseudodoriae*  **sp. nov** | Thailand, Surat Thani Prov., Ko Samui Dist., Ko Samui Island | 2 | ZMKU AM 01584 | B | - | - | This study |
| *L. pseudodoriae*  **sp. nov** | Thailand, Surat Thani Prov., Ko Samui Dist., Ko Samui Island | 2 | ZMKU AM 01585 | A, B | MW567830 | MW574620 | This study |
| *L. pseudodoriae* **sp. nov** | Thailand, Surat Thani Prov., Ko Samui Dist., Ko Samui Island | 2 | ZMKU AM 01586 | B | - | - | This study |
| *L. pseudodoriae*  **sp. nov** | Thailand, Surat Thani Prov., Ko Samui Dist., Ko Samui Island | 2 | ZMKU AM 01587 | B | - | - | This study |
| *L. pseudodoriae*  **sp. nov** | Thailand, Surat Thani Prov., Ko Samui Dist., Ko Samui Island | 2 | ZMKU AM 01589 | B | - | - | This study |
| *L. savan* | Laos, Savannakhet Prov., Vilabouli Dist. | - | NCSM 76287 | A | MK688597 | - | [7] |
| *L. savan* | Laos, Savannakhet Prov., Vilabouli Dist. | - | NCSM 76289 | A | MK688599 | - | [7] |
| *L. savan* | Laos, Savannakhet Prov., Vilabouli Dist. | - | NCSM 76294 | A | MK688601 | - | [7] |
| *L. woodworthi* | Luzon Island., Philippines | - | RMB 4092 | A | JX911331 | - | [11] |

References

1. Liu, Z.Q.; Wang, Y.Q.; Su, B. The mitochondrial genome organization of the rice frog, *Fejervarya limnocharis* (Amphibia: Anura): a new gene order in the vertebrate mtDNA. *Gene* **2005**, *346*, 145−151
2. Zhou, Y.; Zhang, J.Y.; Zheng, R.Q.; Yu, B.G.; Yang, G. Complete nucleotide sequence and gene organization of the mitochondrial genome of *Paa spinosa* (Anura: Ranoidae). *Gene* **2009**, *447*, 86–96.
3. Zhang, J.F.; Nie, L.W.; Wang, Y.; Hu, L.L. The complete mitochondrial genome of the large-headed frog, *Limnonectes bannaensis* (Amphibia: Anura), and a novel gene organization in the vertebrate mtDNA. *Gene* **2009**, *442*, 119–127.
4. Inger, R.F.; Stuart, B.L. Systematics of *Limnonectes* (*Taylorana*) Dubois. *Curr. Herpetol.* **2010**, *29*, 51−86.
5. Phimmachak, S.; Sivongxay, N.; Saeteun, S.; Yodthong, S.; Rujirawan, A.; Neang, T.; Aowphol, A.; Stuart, B.L. A new *Limnonectes* (Anura: Dicroglossidae) from southern Laos. *Zootaxa* **2018**, *4375*, 325–340.
6. Chen, L.; Murphy, R.W.; Lathrop, A.; Ngo, A.; Orlov, N.L.; Ho, C.T.; Somorjai, I.L.M. Taxonomic chaos in Asian ranid frogs: An initial phylogenetic resolution. *Herpetol. J.* **2005**, *15*, 231–243
7. Phimmachak, S.; Richards, S.J.; Sivongxay, N.; Saeteun, S.; Chuaynkern, Y.; Makchai, S.; Som, H.E.; Stuart, B.L. A caruncle-bearing fanged frog (*Limnonectes*, Dicroglossidae) from Laos and Thailand. *Zookeys* **2019**, *846*, 133–155.
8. Evans, B.J.; Brown, R.M.; McGuire, J.A.; Supriatna, J.; Andayani, N.; Diesmos, A.; Iskandar, D.; Melnick, D.J.; Cannatella, D.C. Phylogenetics of fanged frogs: Testing biogeographical hypotheses at the interface of the Asian and Australian faunal zones. *Syst. Biol.* **2003**, *52*, 794−819.
9. Matsui, M.; Nishikawa, K.; Eto, K. A new burrow-utilising fanged frog from Sarawak, East Malaysia (Anura: Dicroglossidae). *Raffles Bull. Zool.* **2014**, *62*, 679–687.
10. Aowphol, A.; Rujirawan, A.; Taksintum, W.; Chuaynkern, Y.; Stuart, B.L. A new caruncle-bearing *Limnonectes* (Anura: Dicroglossidae) from northeastern Thailand. *Zootaxa* **2015**, *3956*, 258–270.
11. Oaks, J.R.; Sukumaran, J.; Esselstyn, J.A.; Linkem, C.W.; Siler, C.D.; Holder, M.T.; Brown, R.M. Evidence for climate-driven diversification? A caution for interpreting abc inferences of simultaneous historical events. *Evolution* **2013**, *67(4)*, 991−1010.
12. Matsui, M.; Nishikawa, K. Description of a new species of *Limnonectes* from Sarawak, Malaysian Borneo (Dicroglossidae, Anura). *Curr. Herpetol.* **2014**, *33(2)*, 135−147.
13. Lambertz, M.; Hartmann, T.; Walsh, S.; Geissler, G.; McLeod, D.S. Anatomy, histology, and systematic implications of the head ornamentation in the males of four species of *Limnonectes* (Anura: Dicroglossidae). *Zool. J. Linn. Soc.* **2014**, *172*, 117–132.
14. Frost, D.R.; Grant, T.; Faivovich, J.; Bain R.H.; Haas A.; Haddad C.F.B.; de Sá R.O.; Channing A.; Wilkinson M.; Donnellan S.C.; Raxworthy C.J.; Campbell, J.A.; Blotto, B.L.; Moler, P.; Drewes, R.C.; Nussbaum, R.A.; Lynch, J.D.; Green, D.M.; Wheeler, W.C. The amphibian tree of life. *Bull. Am. Mus. Nat. Hist.* **2006**, *297*, 1–370.
