# Supplementary material for: A New Limnonectes (Anura: Dicroglossidae) from Southern Thailand
Source: Animals (Basel). 2021 Feb 22;11(2):566. doi: 10.3390/ani11020566 (PMC7926908; doi:10.3390/ani11020566)
Supplement: Supplementary file 1 [file animals-11-00566-s001.zip › animals-1097152/animals-1097152-supplementary/Supplement_Table S2.docx]

**Table S2.** Morphological measurements (mm) of adult male specimens of *Limnonectes pseudodoriae* **sp. nov.** and *L. doriae*. Data are given as mean and standard deviation, followed by range in parentheses.

| **Characters** | ***L. pseudodoriae* sp. nov.** | | ***L. doriae*** | |
| --- | --- | --- | --- | --- |
|  | Holotype  ZMKU AM 01567 | Paratypes | Myanmar | Thailand |
|  | *n* = 1 | *n* = 17 | *n* = 1 | *n* = 15 |
| SVL | 47.3 | 45.1 ± 1.6 (42.6−48.2) | 52.5 | 47.5 ± 3.6  (41.4−55.0) |
| HDL | 23.4 | 21.4 ± 1.2  (20.0−23.5) | 20.7 | 22.0 ± 2.2  (18.5−25.4) |
| HDW | 24.0 | (21.5 ± 1.4  (19.0−24.1) | 22.7 | 21.6 ± 2.2  (18.1−25.6) |
| SNT | 9.3 | 8.2 ± 0.4  (7.3−9.0) | 9.1 | 8.3 ± 0.8  (7.1−10.0) |
| EYE | 4.7 | 4.7 ± 0.3  (4.1−5.1) | 5.2 | 5.0 ± 0.4  (4.2−5.8) |
| IOD | 5.5 | 4.7 ± 0.5  (3.9−5.4) | 6.3 | 5.0 ± 0.7  (3.7−6.0) |
| IND | 4.1 | 3.7 ± 0.3  (3.3−4.6) | 5.6 | 4.8 ± 0.6  (3.7−5.8) |
| SHK | 24.2 | 22.7 ± 1.0  (20.9−24.8) | 29.9 | 24.8 ± 2.1  (20.6−27.4) |
| TGH | 24.2 | 23.8 ± 1.0  (22.0−25.6) | 27.8 | 25.9 ± 1.8  (21.8−28.2) |
| LAL | 9.4 | 9.9 ± 0.5  (9.2−10.7) | 10.1 | 9.6 ± 1.0  (7.6−11.0) |
| HND | 11.8 | 11.1 ± 0.6  (9.7−12.1) | 13.2 | 11.5 ± 0.9  (9.8−12.6) |
| FTL | 23.7 | 22.5 ± 1.0  (20.9−24.6) | 27.0 | 24.8 ± 1.8  (20.6−26.9) |
| IML | 2.8 | 3.0 ± 0.2  (2.6−3.3) | 4.4 | 3.6 ± 0.4  (2.9−4.2) |
| IMW | 1.3 | 1.2 ± 0.2  (0.9−1.6) | 1.7 | 1.4 ± 0.3  (0.9−1.9) |
| TMP | 7.0 | 5.7 ± 0.8  (4.2−6.8) | 4.6 | 5.1 ± 0.7  (4.0−6.1) |
| TMP/EYE | 1.49 | 1.23 ± 0.17  (0.82−1.48) | 0.89 | 0.75−1.19  (1.02 ± 0.13) |
