# Supplementary material for: A New Limnonectes (Anura: Dicroglossidae) from Southern Thailand
Source: Animals (Basel). 2021 Feb 22;11(2):566. doi: 10.3390/ani11020566 (PMC7926908; doi:10.3390/ani11020566)
Supplement: Supplementary file 1 [file animals-11-00566-s001.zip › animals-1097152/animals-1097152-supplementary/Supplement_Table S3.docx]

**Table S3.** Morphological measurements (mm) of adult female specimens of *Limnonectes pseudodoriae* **sp. nov.** and *L. doriae*. Data are given as mean and standard deviation, followed by range in parentheses.

| **Characters** | ***L. pseudodoriae* sp. nov.** | ***L. doriae*** | |
| --- | --- | --- | --- |
|  | Paratypes | Paratype  FMNH 97974 | Thailand |
|  | *n* = 14 | *n* = 1 | *n* = 13 |
| SVL | 40.0 ± 2.8  (36.0−44.1) | 39.4 | 45.4 ± 3.0  (41.4−50.3) |
| HDL | 16.7 ± 0.9  (15.4−18.1) | 15.7 | 18.6 ± 1.1  (16.6−20.1) |
| HDW | 15.9 ± 1.0  (14.1−17.5) | 17.2 | 17.9 ± 1.0  (15.9−17.9) |
| SNT | 6.5 ± 0.4  (5.8−7.1) | 6.9 | 7.5 ± 0.5  (6.8−8.2) |
| EYE | 4.3 ± 0.3  (4.0−5.0) | 5.2 | 4.9 ± 0.3  (4.2−5.3) |
| IOD | 3.3 ± 0.2  (2.9−3.7) | 3.6 | 3.9 ± 0.4  (3.5−4.6) |
| IND | 3.2 ± 0.4  (2.6−3.8) | 4.9 | 4.3 ± 0.4  (3.5−4.9) |
| SHK | 20.0 ± 1.3  (17.5−22.4) | 22.4 | 24.0 ± 0.9  (22.5−25.3) |
| TGH | 21.6 ± 1.4  (19.4−24.1) | 20.7 | 25.1 ± 1.4  (23.0−26.7) |
| LAL | 9.0 ± 0.7  (8.1−10.4) | 8.6 | 9.5 ± 0.4  (8.8−10.2) |
| HND | 9.9 ± 0.8  (8.8−11.3) | 8.5 | 10.9 ± 0.6  (9.9−12.8) |
| FTL | 20.1 ± 1.4  (18.1−22.6) | 20.9 | 24.2 ± 1.2  (21.7−26.1) |
| IML | 2.4 ± 0.2  (2.1−2.9) | 3.0 | 3.5 ± 0.3  (3.2−4.0) |
| IMW | 1.0 ± 0.1  (0.9−1.2) | 1.1 | 1.2 ± 0.2  (0.9−1.5) |
| TMP | 3.4 ± 0.2  (3.0−3.8) | 2.6 | 3.5 ± 0.2  (3.1−4.0) |
| TMP/EYE | 0.79 ± 0.06  (0.69−0.93) | 0.51 | 0.72 ± 0.07  (0.62−0.82) |
