# Supplementary material for: A New Limnonectes (Anura: Dicroglossidae) from Southern Thailand
Source: Animals (Basel). 2021 Feb 22;11(2):566. doi: 10.3390/ani11020566 (PMC7926908; doi:10.3390/ani11020566)
Supplement: Supplementary file 1 [file animals-11-00566-s001.zip › animals-1097152/animals-1097152-supplementary/Supplement_Table S4.docx]

**Table S4.** Call parameters of three male paratypes of *Limnonectes pseudodoriae* **sp. nov.** Parameter values are given as mean ± SD and ranges in parentheses.

| **Call parameters** | **Paratype**  **ZMKU AM 01579**  *n*_call_ = 60 | **Paratype**  **ZMKU AM 01580**  *n*_call_ = 60 | **Paratype**  **ZMKU AM 01581**  *n*_call_ = 60 |
| --- | --- | --- | --- |
| Call duration (ms) | 251.56 ± 169.56  (126.35–1,288.82) | 311.80 ± 280.41  (124.56–1,140.48) | 443.62 ± 459.00  (134.65–1,965.07) |
| Intercall interval (s) | 1.02 ± 1.16  (0.38–8.51) | 1.52 ± 1.87  (0.48–10.84) | 1.30 ± 1.43  (0.42–7.91) |
| Call rate (call/min) | 46.39 | 32.46 | 34.09 |
| Number of Note (note) | 1.62 ± 0.88  (1–7) | 1.88 ± 1.38  (1–6) | 2.45 ± 2.20  (1–10) |
| Note duration (ms) | 135.47 ± 8.39  (121.95–163.34) | 135.80 ± 12.23  (117.65–163.29) | 148.55 ± 12.46  (131.33–183.86) |
| Internote interval (ms) | 37.45 ± 10.48  (14.61–72.36) | 49.12 ± 8.43  (33.64–64.55) | 39.45 ± 12.87  (7.97–75.05) |
| Note rate (notes/s) | 5.91 ± 0.44  (4.95–7.11) | 5.48 ± 0.38  (4.97–6.14) | 5.44 ± 0.43  (4.55–6.23) |
| Dominant frequency (kHz) | 0.90 ± 0.10  (0.60–0.95) | 1.00 ± 0.04  (0.95–1.03) | 0.78 ± 0.12  (0.52–0.86) |
| Temperature (°C) | 26.0 | 26.1 | 26.0 |
